# Topological packing statistics of living and nonliving matter

**DOI:** 10.1126/sciadv.adg1261

**Published:** 2023-09-06

**Authors:** Dominic J. Skinner, Hannah Jeckel, Adam C. Martin, Knut Drescher, Jörn Dunkel

**Affiliations:** ^1^ Department of Mathematics, Massachusetts Institute of Technology, 77 Massachusetts Avenue, Cambridge, MA 02139, USA.; ^2^ NSF-Simons Center for Quantitative Biology, Northwestern University, 2205 Tech Drive, Evanston, IL 60208, USA.; ^3^ Department of Physics, Philipps-Universität Marburg, Renthof 6, 35032 Marburg, Germany.; ^4^ Biozentrum, University of Basel, Spitalstrasse 41, 4056 Basel, Switzerland.; ^5^ Department of Biology, Massachusetts Institute of Technology, 77 Massachusetts Ave., Cambridge, MA 02139, USA.

## Abstract

Complex disordered matter is of central importance to a wide range of disciplines, from bacterial colonies and embryonic tissues in biology to foams and granular media in materials science to stellar configurations in astrophysics. Because of the vast differences in composition and scale, comparing structural features across such disparate systems remains challenging. Here, by using the statistical properties of Delaunay tessellations, we introduce a mathematical framework for measuring topological distances between general three-dimensional point clouds. The resulting system-agnostic metric reveals subtle structural differences between bacterial biofilms as well as between zebrafish brain regions, and it recovers temporal ordering of embryonic development. We apply the metric to construct a universal topological atlas encompassing bacterial biofilms, snowflake yeast, plant shoots, zebrafish brain matter, organoids, and embryonic tissues as well as foams, colloidal packings, glassy materials, and stellar configurations. Living systems localize within a bounded island-like region of the atlas, reflecting that biological growth mechanisms result in characteristic topological properties.

## Teaser

A topological metric framework makes it possible to compare biological, physical and astronomical systems.

## INTRODUCTION

Topology (*1*) studies the fundamental neighborhood relations among living (*2*–*4*), nonliving (*5*, *6*), or abstract (*7*, *8*) entities. By ignoring object-specific features, such as particle shape or chemical composition, topological analysis of spatial packings and nearest-neighbor networks can reveal universal ordering principles (*9*, *10*) that extend across broad classes of systems (*11*). As recent advances in high-resolution imaging (*12*, *13*), deep learning–based image analysis (*14*), and simulation (*15*–*18*) techniques are offering unprecedented insights into the spatial organization of biological (*19*–*21*) and physical (*22*) matter, there now exists a unique opportunity to explore the topological similarities and differences across a diverse range of complex systems, from bacterial communities and eukaryotic tissues to amorphous materials (*23*) or large-scale astrophysical structures (*24*). A central open question in this context is whether, or to which extent, living and nonliving matter have distinct topological properties.

**Fig. 1.**
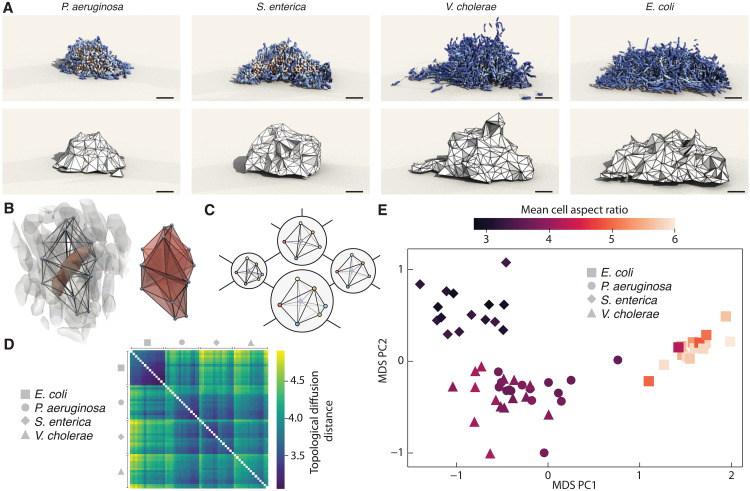
Topological diffusion distance distinguishes prokaryotic multicellular colonies from local topological information alone. (**A**) 3D reconstruction of bacterial biofilms formed by the species *P. aeruginosa*, *S. enterica*, *V. cholerae*, and *E. coli* [top row; ~2000 cells per biofilm; experimental data from (*32*)]. The topological structure is encoded in (the α-complex of) the Delaunay tessellation (bottom row; section S1). Color indicates local density (red, high; blue, low). Scale bar, 5 μm. (**B**) For each cell (red, left), we determine its nearest-neighbor motif (right), formed by all Delaunay tetrahedrons that have the cell and its neighbors as vertices (centroids of neighboring cells shown as blue spheres). (**C**) Motifs only change through discrete topological transitions (flips), which naturally induces a graph structure where each vertex is a motif and vertices are connected if they are one transition apart, illustrated here for selected motifs. Vertex size reflects the relative frequency of a motif. Each biofilm is thus mapped to a probability distribution of motifs over this flip graph. (**D**) Pairwise topological diffusion distance (TDD) matrix between all 4 × 15 experiments, grouped by species. The block structure shows that the TDD detects differences between the species. (**E**) Topological atlas obtained from the MDS embedding of the TDD distance matrix, with the first two principal components (PC) shown. The embedding is colored by the mean cell aspect ratio, showing that topological changes correlate strongly with changes in the cell geometry. TDD identifies every pair of biofilm species as statistically different at *P* < 0.01 (fig. S11).

**Fig. 2.**
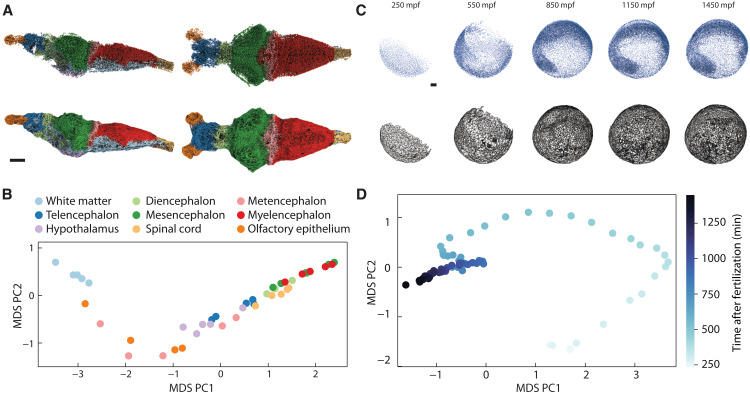
Topological statistics distinguish static and dynamic eukaryotic architectures. (**A**) Nuclei within a juvenile zebrafish brain, as measured with x-ray microtomography, colored by nine major brain regions (top), with corresponding α-complex of Delaunay tessellation (bottom). Data are from (*13*). Scale bar, 100 μm. (**B**) Topological analysis detects systematic differences between regions of the zebrafish brain (see also fig. S12), revealing that brain tissue architectures vary along a 1D topological manifold. The topological distance was computed pairwise between nine brain regions across five separate experiments, and the resulting distance matrix was embedded with MDS. Although overall brain size and morphology differs across the experiments, corresponding regions of the brain lie together in the embedding space. (**C**) Cell nuclei in a zebrafish embryo imaged during development with light-sheet microscopy (top) and the corresponding α-complex of Delaunay tessellation (bottom). Data are from (*19*). Scale bar, 200 μm [minutes post fertilization (mpf)]. (**D**) Collecting the average topological distribution at 90 different time points and computing the pairwise distance matrix, the resulting MDS embedding recovers a curve parameterized by time. Note that, although the distance calculation and embedding are not explicitly aware of the temporal ordering of the data, the developmental progression is imprinted in the tissue topology and can thus be recovered from the TDD matrix.

**Fig. 3.**
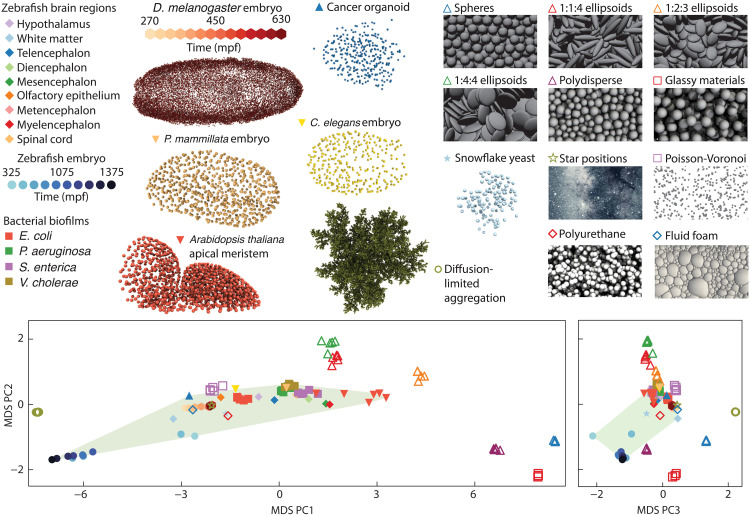
Combined topological atlas reveals topological variation across living and nonliving systems. The atlas was constructed by calculating the TDD between various biological and physical systems: bacterial biofilms, zebrafish brain regions, zebrafish embryo, fly embryo (*Drosophila melanogaster*), worm (*Caenorhabditis elegans*) embryo, sea squirt embryo (*Phallusia mammillata*), human cancer organoid, plant (*Arabidopsis thaliana*) apical meristem, snowflake yeast, simulated random packings, a polyurethane industrial foam, a simulated fluid foam, a diffusion-limited aggregation model, Poisson-Voronoi tessellations, and a collection of stars close to Earth (table S1). The first three principal components of the MDS embedding are shown, using filled symbols for living systems and nonfilled symbols for nonliving systems. For the various datasets analyzed here, the convex hull enclosing the first three MDS components of the living systems (green) contains the industrial foam as the only nonliving systems (1 of 40 nonliving points).

To address this problem, we introduce here a general mathematical framework for comparing the topology of three-dimensional (3D) disordered packings (*6*) or, more generally, point clouds. These point clouds can represent the positions of the cell nuclei in a piece of tissue (*12*, *25*, *26*), the midpoints of bacteria within a biofilm (*27*, *28*), the atoms in a liquid or solid (*23*, *29*), or the loci of nearby stars in our galaxy (*30*). Extending recent progress in the statistical characterization of topological structures (*6*, *29*, *31*), we developed a computationally efficient algorithm that makes it possible to directly compare all these and many other systems, without requiring curated training data. Intuitively, the underlying numerical scheme has two steps: In the first step, the algorithm determines the relative frequencies of typical neighborhood patterns (“motifs”) within a given 3D point cloud; in the second step, it computes the cost of transforming one motif frequency distribution into another by exploiting a natural graph structure on the space of motifs. By applying this framework to a diverse set of experimental and simulated data, we find that basic topological information suffices to identify biofilms from different bacterial species (Fig. 1), to determine time ordering and developmental transitions in zebrafish embryos (Fig. 2), and to distinguish these and other living systems from a variety of inanimate physical structures (Fig. 3). More generally, the statistical analysis approach developed here opens a path toward quantifying and comparing the structural differences within and across broad classes 3D disordered media.

## RESULTS

### Topological representation of disordered systems

Our starting point for constructing a metric framework that can measure distances between 3D disordered structures is the classical Delaunay tessellation (*9*), which is a topological object linking nearest neighbors in a point cloud (Materials and Methods). In three spatial dimensions, Delaunay tessellations are composed of adjacent tetrahedrons, illustrated in Fig. 1A for bacterial biofilm imaging data from four different species (*32*). If we pick any cell within a biofilm, then its nearest neighbors form an elementary motif consisting of the corresponding Delaunay tetrahedrons (Fig. 1B). In a disordered multicellular system, these nearest-neighbor motifs typically differ from cell to cell, but, as we show in section S2 and fig. S4, it is possible to uniquely identify and label each possible motif. This important fact allows us to count how often each motif appears within the bulk of a given 3D structure. To distinguish the bulk material from the boundary, which can have different topological properties (section S1), we use the α-complex (*33*), an interior subset of the Delaunay tessellation (Materials and Methods and section S1).

Having captured the topological properties of a material as a distribution over neighborhood motifs, the second important observation is that one can determine how many elementary neighbor exchanges (“flips”) are needed to transform one motif into another (section S2). This means that we can construct a so-called “flip graph” (*31*) where each vertex represents a specific motif and edges link motifs that are exactly one flip apart from each other (Fig. 1C). Any discrete 3D material structure can then be identified with a specific empirical probability distribution on the flip graph, by assigning to every motif its relative frequency in the material, indicated by the relative vertex size in Fig. 1C. In practice, a few hundred cells or particles often suffice to obtain a sufficiently accurate approximation of the empirical motif probability distributions on the flip graph (section S3).

### Topological distance between motif distributions

With the weighted flip graph representation at hand, measuring the topological distance between two disordered structures reduces to comparing their associated probability distributions on the flip graph. To do so, we implement a topological diffusion distance (TDD) which relaxes the well-known earth mover’s distance (*31*), or optimal transport, on a graph (Materials and Methods and section S3), and use the TDD to compare structures of biological and nonbiological matter. Intuitively, such transport distances measure how probability density needs to be shuffled along the flip graph to transform one motif distribution into another. By using information about the separation of motifs on the flip graph, these transport distances are better at distinguishing disordered structures than conventional entropic distance measures (section S3). The TDD has the practical advantage over other distances, that it can be efficiently computed, enabling a fast comparison of 3D structures even with several million points and thousands of motifs.

### Topological differences between bacterial biofilms

As a first application, we find that the TDD is able to detect the subtle topological differences between biofilms formed by the bacterial species *Pseudomonas aeruginosa*, *Salmonella enterica*, *Vibrio cholerae*, and *Escherichia coli* [Fig. 1, D and E; 15 biofilm colonies per species with ∼2000 cells each, data from (*32*)]. We used the 3D spatial position of cell centroids to compute a pairwise TDD distance matrix between colonies. The block structure of the distance matrix (Fig. 1D), and its 2D embedding obtained using multidimensional scaling (MDS) (*34*) (Fig. 1E), show that the topological information encoded in the Delaunay tessellations suffices to distinguish the 3D structure of these four prokaryotic systems. In particular, we observe a close correlation between the mean cell aspect ratio and the topological clustering in the MDS atlas, which illustrates that the TDD can detect fine differences in positional and orientational ordering arising from the interplay of steric repulsion and growth memory in bacterial biofilms (*27*, *28*, *32*, *35*).

### Topological differences between zebrafish brain matter

To demonstrate the practical potential of the TDD framework for characterizing both static and dynamic tissue architecture in more complex eukarotic organisms, we next analyze the topology of brain and embryo tissues. State-of-the-art microscopy and image analysis methods are able to visualize and segment the cell nuclei within the brains of commonly studied model organisms (*13*, *14*). An open question is whether spatially and functionally distinct brain regions have different topological characteristics and, if so, what the properties of the underlying topological manifold are. As a specific example, we consider the juvenile zebrafish brain, for which ∼80,000 nuclei positions were recently measured (*13*) with x-ray microtomography (Fig. 2A). Computing the TDD matrix (fig. S14), and its 2D MDS embedding (Fig. 2B), for nine different brain regions across 5 experiments (*13*) reveals substantial topological differences between the white matter, hypothalamus, and diencephalon regions. However, all nine brain regions localize near a 1D manifold (Fig. 2B), suggesting that topological variability in the juvenile zebrafish brain tissue is highly constrained and can be effectively described by a single differentiation parameter along this manifold (*36*).

### Topological changes during multicellular development

In addition to comparing static multicellular structures, the TDD makes it possible to quantify the topological changes of tissues during embryonic development. Recent advances in light-sheet microscopy enable the nondestructive imaging of tissue dynamics at a fine temporal resolution (*12*, *19*). An open question is whether topological information suffices to detect and define structural transitions during embryogenesis. Taking zebrafish development as a widely studied example, we analyzed light-sheet microscopy data from (*19*), which reported the positions of all cell nuclei from around 200 to 1500 min post fertilization (mpf), during which the number of cells increases from a few hundreds to around 15,000. As cells divide and rearrange to form different tissue domains, the internal structure of the Delaunay tessellations changes in time. Five snapshots showing the evolution of the Delaunay networks of a zebrafish embryo are shown in Fig. 2C; in total, 900 time points were imaged at regular intervals separated by 90s. Dividing the data into 90 time intervals, each containing 10 sequential time points, we computed the 90 × 90 TDD matrix (fig. S15). The planar MDS embedding shows the topological trajectory of the zebrafish embryo (Fig. 2D). Note that, even though the TDD does not explicitly use temporal information, one can recover the temporal ordering of the imaging data by following the topological trajectory reconstructed from the TDD. In other words, the nonequilibrium temporal growth trajectory is ingrained into the Delaunay structure. Therefore, changes in the topological trajectory can be used to define an intrinsic topological clock of the embryo, reminiscent of the proper times derived from the particle world lines in special and general relativity (*37*).

An interesting mathematical aspect of the topological distance framework, with substantial future theoretical and practical potential, is that the TDD provides a foundation for developing a comprehensive geometric characterization of topological trajectories (as in Fig. 2, B and D) as well as higher-dimensional embedding manifolds (such as in Fig. 1E). This can be achieved combining the TDD approach with known ideas and results from distance geometry (*38*). Intuitively, distance geometry makes it possible to introduce well-defined notions of curvature and other geometric concepts (section S4) on abstract point sets endowed with a metric structure; for instance, in Fig. 2D, each point represents an ensemble of Delaunay tessellations (developmental states of the zebrafish embryo) and a metric is provided by the TDD. By measuring the distances between three nearby points along the topological trajectory in Fig. 2D, one can compute the local Menger curvature of the developmental trajectory, corresponding to the inverse radius of the circumcircle of the three points (fig. S8). An application to zebrafish data (*19*) suggests that regions of extremal topological curvature correlate with structural transitions during biological development (section S4).

### Topological comparison of living and nonliving systems

Arguably one of the most interesting applications of the TDD framework concerns our initial question: Are the topological architectures of living systems typically distinct from those of nonliving systems? Because the TDD does not require system-specific information beyond 3D Delaunay tessellations, it can be used to compute the topological distance between any pair of systems for which such tessellations are available. To initiate a cross-disciplinary comparison, we determined pairwise TDDs between various biological and physical systems (section S6 and table S1), including bacterial biofilms (*32*), embryonic tissues from zebrafish (*19*), worms (*39*), sea squirts (*40*), flies (*12*), human cancer organoids (*41*), snowflake yeast (*10*), plant shoot tips (*42*), random Poisson-Voronoi point sets, diffusion-limited aggregation structures, simulated granular packings (*22*), fluid and industrial foams (*43*, *44*), and stars in our galaxy (*30*). The resulting combined topological atlas (3D MDS embedding) in Fig. 3 suggests that the topological architectures of biological systems are typically distinct from those of many nonliving physical systems. Of 40 points corresponding to nonliving systems, only an industrial foam is contained within the convex hull of points in the MDS corresponding to living systems.

Which common structural features present in living systems give rise to their topological similarity? Contributing factors may include the structure induced by growth, a particular distribution of cell geometries, or a common soft cell-cell interaction mechanism in contrast to the hard excluded volume interaction of the granular packings. We can isolate the effect of structure induced by growth, through randomizing cell centroids, which removes the growth memory while retaining cell geometry and soft cell-cell interactions. Specifically, we take bulk data from the zebrafish brain, permute the spatial positions of cells, and then allow the system to relax through cell-cell interactions, resulting in a random packing with the same cell geometry as the original living system (section S7). We find that this randomization leads to systematically different topological packings, more similar to that of the polyurethane foam (fig. S21). This demonstrates that the mechanism by which a system forms has a measurable impact on the topology.

## DISCUSSION

The above results illustrate how the topological metric framework can be used to analyze and compare 3D disordered systems within and across disciplines, from microbiological to astrophysical scales. In particular, the statistical properties of Delaunay tessellations suffice to distinguish representative prokaryotic and eukaroytic multicellular packings from a wide variety of ordered and disordered physical structures. Our analysis of naturally grown and randomized cell packings showed that structural memory, arising from positional and orientational correlations inherited during cell division and growth,

contributes to characteristically different neighborhood motif distributions in biological systems. This conclusion is supported by recent live-imaging experiments that demonstrated the importance of growth-induced ordering in bacterial (*27*, *28*, *32*, *35*, *45*) and eukaryotic systems (*46*).

In the future, the topological distance framework introduced here can help overcome major current challenges in the analysis of natural and engineered multicellular structures, from quantifying how genetic mutations, diseases, and drugs modify tissue architectures (*47*) to the evaluation and classification of tumors (*48*) and organoids (*41*, *49*). Unlike machine learning approaches, which are biased by the choice of training data and may fail to generalize (*50*), the topological approach requires no training data, only the data to be compared. As more and more high-resolution 3D imaging data become available in the next years, they can be added to the topological atlas (Fig. 3) through the online platform (*51*) provided with this work. A particularly interesting challenge will be the identification and characterization of other exceptional biological or physical systems that cross the topological boundaries between living and nonliving matter.

## MATERIALS AND METHODS

### Delaunay tessellation

Given a set of points, *X* = {***x****_i_*}, with ***x****_i_* ∈ ℝ^3^, the Voronoi diagram divides space into polygonal regions *V_i_* associated to each point, ***x****_i_*

$$V_{i}=\{y|\|x_{i}-y\|\leq \|x_{j}-y\|\forall x_{j}\in X\}$$ (1)

The Delaunay tessellation, DT (*X*), is dual to the Voronoi diagram; two points are connected in the Delaunay tessellation if their regions of the Voronoi diagram share a face. More generally, the Delaunay tessellation is a simplicial complex, specified by a set of tetrahedrons in 3D (or triangles in 2D) (*52*), with each tetrahedron defined by four points in *X*, for instance *T* = {***x****_i_*, ***x****_j_*, ***x****_k_*, ***x****_l_*}.

### Motifs

A point ***x**_i_* is a neighbor of a point ***x****_j_* if there exists a tetrahedron *T* with ***x****_i_*, ***x****_j_* ∈ *T*. A neighborhood (of radius 1) *N*(***x****_i_*) is the set of all neighbors of ***x****_i_*. We define the motif, *M_i_*, at point ***x****_i_* as the simplicial complex, and subcomplex of the full Delaunay, which contains all tetrahedrons *T* such that *T* ⊆ {***x****_i_*} ∪ *N*(***x****_i_*). The exact topological information contained in the motif can be stored in a vector after a canonical labeling (section S2). In practice, we have observed more than 300,000 unique motifs, providing a rich topological space to characterize disordered material. Taking instead a neighborhood of radius 2 or higher (*31*) would massively increase the number of motifs, rendering practical computations unfeasible (section S1).

### Boundary points and the α-complex

Points on the boundary of a material can have different topological properties than points in the bulk. We define a point ***x****_i_* as being a boundary point if ∃ ***y*** with ‖***x****_i_* − ***y***‖ = α and ‖***x****_j_* − ***y***‖ ≥ α for all other points ***x****_j_*, i.e., if a sphere of radius α, can touch a point ***x****_i_* while containing no other points, then ***x****_i_* is a boundary point, where α is a parameter that we choose depending on the data (section S1 and table S1). This definition relates to the α-complex of the Delaunay tessellation (*33*) (section S1).

### Motif distributions and the flip graph

We define the topological distribution of a material as the probability distribution over the space of motifs. Motifs only change through discrete topological transitions, and this defines a natural graph structure over the space of motifs. Specifically, the flip graph is the graph where two motifs (vertices) are connected by an edge if one motif can be transformed into the other by a single topological transition.

### Topological diffusion distance

We wish to compare distributions ρ_A_, ρ_B_, for materials A and B, which live over the space of motifs, while making use of the flip graph structure. To do so, we first define the directed incidence matrix for the flip graph

$$D_{ev}=\left\{ \begin{array}{c} 1\;\mathrm{if}\;\exists \;w\;\mathrm{with}\;e=(v,w)\\ -1\;\mathrm{if}\;\exists \;w\;\mathrm{with}\;e=(w,v)\\ 0\;\mathrm{else} \end{array}\right.$$ (2)

where *e* are graph edges, and *v* and *w* are graph vertices. From this, we define the graph Laplacian *L* = *D*^⊤^*D*. The TDD is defined as

$$d_{\mathrm{TDD}}(A,B)={||DL^{+}(\rho_{A}-\rho_{B})||}_{1}$$ (3)

where *L*^+^ is the pseudo-inverse. For diffusion interpretation and relationship to optimal transport, see section S3.

## Supplementary Materials

### This PDF file includes:

Supplementary Text Figs. S1 to S21 Table S1 References
