# Supplementary material for: Topological packing statistics of living and nonliving matter
Source: Sci Adv. 2023 Sep 6;9(36):eadg1261. doi: 10.1126/sciadv.adg1261 (PMC10482333; doi:10.1126/sciadv.adg1261)
Supplement: Supplementary file 1 — Supplementary Text Figs. S1 to S21 Table S1 References [file sciadv.adg1261_sm.pdf]

Science Advances
AAAS

Supplementary Materials for

Topological packing statistics of living and nonliving matter

Dominic J. Skinner *et al.*

Corresponding author: Jörn Dunkel, dunkel@mit.edu

Sci. Adv. **9**, eadg1261 (2023)
DOI: 10.1126/sciadv.adg1261

This PDF file includes:

Supplementary Text
Figs. S1 to S21
Table S1
References

I. TOPOLOGICAL DESCRIPTION

A. Delaunay tessellation

Our starting point is a set of points, $X = \{x_i\}$, with $x_i \in \mathbb{R}^3$, with which we want to extract topological information from. Here, we do so by using the Delaunay tessellation, the dual of the Voronoi diagram. Recall that the Voronoi diagram divides space into regions associated with each point, x_i, so that the ith region is the set $V_i = \{y \mid ||y-x_i||_2^2 \leq ||y-x_j||_2^2, \forall j\}$, a definition which can be readily extended for $x_i \in \mathbb{R}^n$. Two points are connected in the Delaunay tessellation if their regions of the Voronoi diagram share a face. Specifically, the Delaunay tessellation of X, $DT(X)$, is a simplicial complex, and is specified by a set of tetrahedrons in 3D (or triangles in 2D). Given any 4 points which form a tetrahedron, $T = \{x_i, x_j, x_k, x_l\}$, the tetrahedron is in the Delaunay complex, $T \in DT(X)$, if the circumsphere of those points contains no other points in X [52]. The resulting simplicial complex generated by those tetrahedrons, is the central object from which we will extract information about our system.

B. Local motifs

Even for two experiment performed under exactly the same conditions, we would not expect the exact same realization of the Delauany tessellation. Instead we seek to characterize the statistical properties of the topological object, and we do so by quantifying local structure. Specifically, we define the local, or egocentric, simplex of radius r around a point as the simplicial complex induced by all simplices consisting of points at most r edges away from the vertex. We also refer to this as a motif, and consider a motif to describe the local neighborhood topology around a point. Every material could then be considered as a probability distribution over the space of motifs, where characterizing the differences between distributions characterizes the topological difference between materials.

The choice of r determines how much information about the neighborhood structure is recorded at each point, but also how many observations are needed to sample the distribution well, and how computationally expensive the numerical problem of calculating the flip graph and distances will be. In 2D, only $O(20)$ distinct motifs of radius $r = 1$ were typically observed and the flip graph was essentially a line graph [31]. In this case $r \geq 2$ was required, and $r = 2$ worked in practice, with some $O(50,000)$ unique motifs observed for $O(700,000)$ points in total [31]. In 3D, even with $r = 1$, $O(300,000)$ unique motifs were observed for some $O(700,000)$ points, and the flip graph is non-trivial. This demonstrates that $r = 1$ provides a large topological space in which to characterize a material. Indeed, a framework using $r = 1$ proves powerful for 3D calculations as demonstrated by the results in the main text and throughout the supplementary information. Taking $r = 2$ would be numerically impractical, as a vast flip graph would be needed. We note that, even at $r = 1$, the large number of unique motifs suggests that we are undersampling the true distribution. Whilst fully sampling the distribution would be preferable, since we have a metric, accurate sampling of every motif is not needed to characterize distributions over the graph at a coarse grained level (see Sec. III D).

In 2D it was equivalent to talk about the graph structure or the simplicial complex. The motifs were near-triangulations, meaning they were planar graphs with one non-triangular face. Taking the non-triangular face as the infinite face is enough to find an embedding, and so determine the simplices. In 3D, the graph structure alone is not enough to determine the simplicial structure, see Fig. S1. This means we could either represent the motifs as the full simplicial complex, or take a more coarse grained approach and just take the graph structure. Here we take the full simplicial complex.

C. Alpha complex

One drawback of the Voronoi diagram is that every point in space (except at boundaries between regions) is assigned to a point. This means that points which are far away from each other in space can end up being neighbours in the Delaunay. For many of the systems considered here, connections between very far away points are unphysical, and such unphysical connections occur at the boundaries of the system, for instance across the surface of the zebrafish embryo, Fig. S2. Since we wish to study the bulk properties of materials, we do not include motifs which reside at boundaries. This is especially relevant if the boundary is artificial. For instance the star survey data only contains the closest stars to earth, which approximately fill out a sphere. The resulting boundary at the edge of this sphere has no physical meaning.

To identify the motifs at the boundary, we work with the alpha-complex of the Delaunay. All tetrahedrons, k, in the Delaunay have a circumsphere with some radius, r_k. The alpha-complex is the simplicial complex made up of all tetrahedrons with circumsphere radius less than some parameter, $r_k < \alpha$ [33]. This introduces a parameter α to be set, which should be chosen to eliminate only the unphysical tetrahedrons of the Delaunay tessellation, whilst

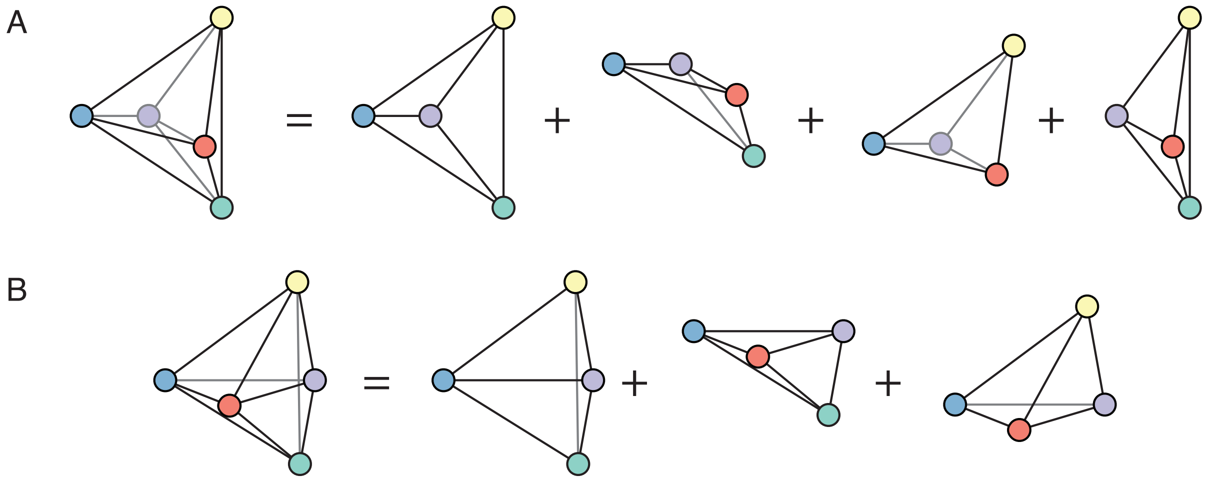

FIG. S1. Example of two different simplicial complexes with the same graph structure. (A) Simplicial complex created out of 4 simplices. (B) A different simplicial complex created from 3 simplices, but with the same graph structure as (A).

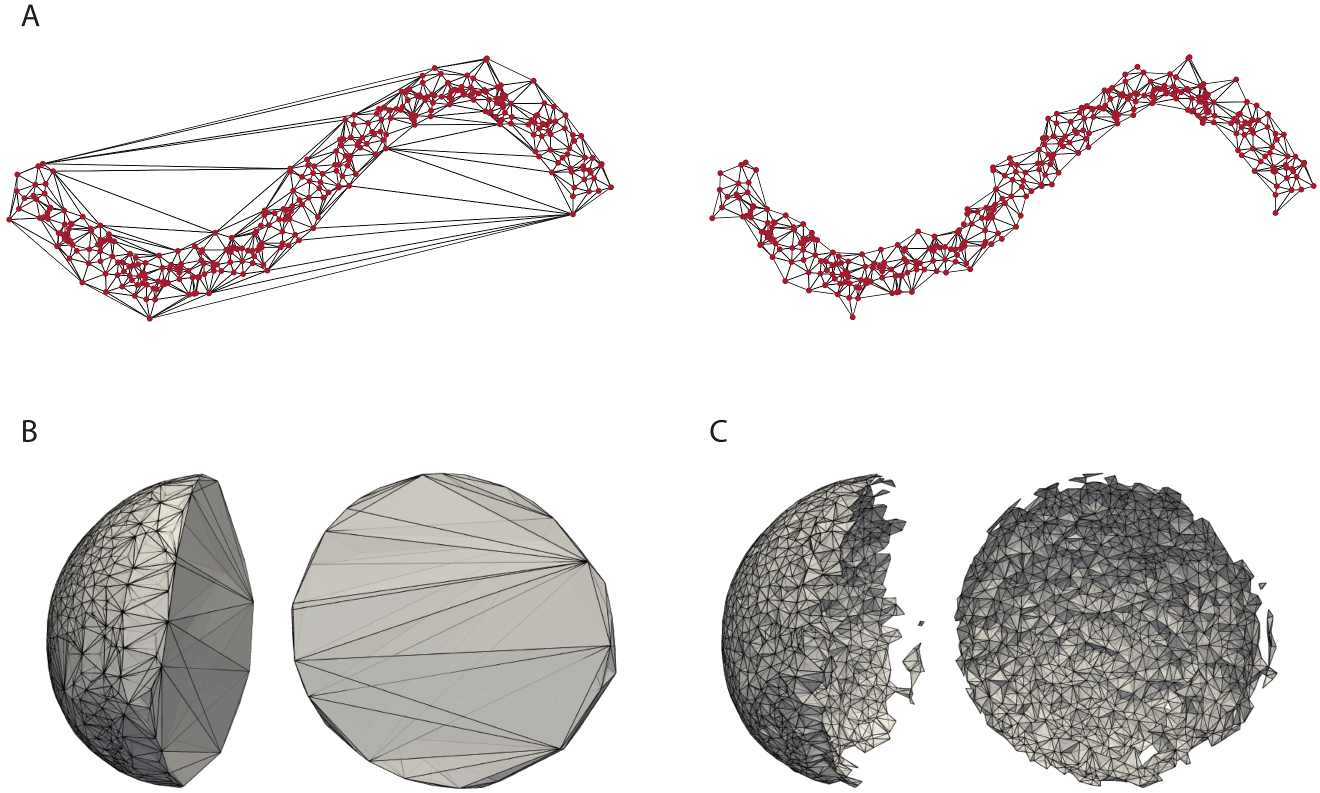

FIG. S2. The alpha complex provides a way to extract a physically motivated simplicial complex from the Delaunay tessellation. (A) The Delaunay tessellation (black lines, left) of some points in $\mathbb{R}^2$ (red dots), contains unphysical connections between far apart points. Taking the alpha complex (black lines, right) by removing triangles with too large of a circumsphere, gives a physically motivated topological structure to work with. (B) For the developing zebrafish embryo at around 300 minutes post fertilization, we see that taking the Delaunay tessellation connects points that are far apart and physically unrelated (two views of the Delaunay tessellation are shown). (C) Taking the alpha complex gives a more physical topological object. Throughout, instead of showing the full Delaunay tessellation we only show the relevant part of it from which we extract motifs, specifically the alpha complex.

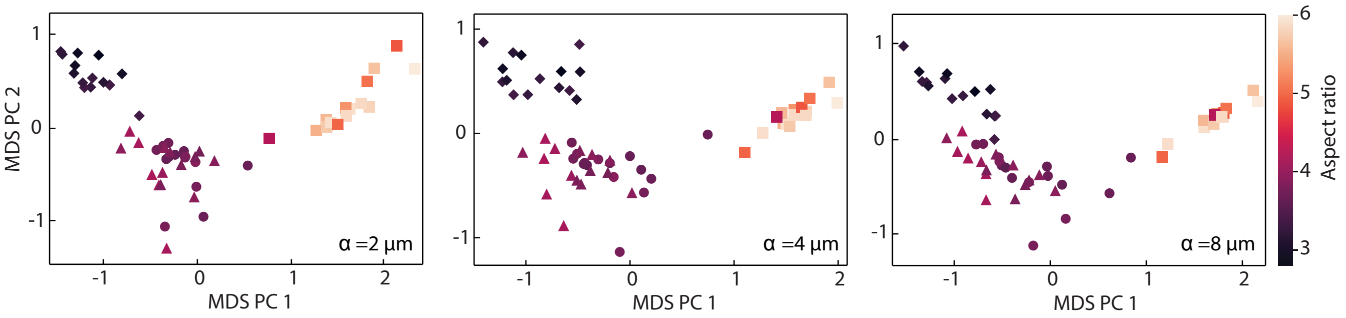

FIG. S3. Topological embeddings are not sensitive to the value of α. In Fig. 1 of the main text, the MDS embedding of different biofilms was computed with $\alpha = 4\mu$m across all experiments. Increasing or decreasing this value by a factor of 2, results in essentially the same embedding. For most systems with more points in the bulk than on the boundary, we do not expect distance calculations to be sensitive on the choice of α.

retaining all others. We find that $\alpha = 2\times$ median r_i typically provides a good choice, although we confirm this by inspecting the 3D point cloud and ensuring that the points identified as being on the boundary really are on the boundary and are not found throughout the bulk. For a particular system, say different biofilm experiments, we fix α across experiments for consistent analysis. The resulting distance calculations should not be overly sensitive on the choice of α, as this only affects a small number of motifs on the boundary of a system. For instance, for the biofilms we took $\alpha = 4\mu$m, although either $\alpha = 2\mu$m or $\alpha = 8\mu$m result in essentially identical embeddings, Fig. S3. Any motif which contains a tetrahedron with circumradius $r_k \geq \alpha$, i.e. too large to be in the alpha complex, is not counted as part of the topological distribution. We use this approach throughout this work, when data is not periodic and so has boundaries, following our previous approach in 2D [31].

Certain 3D systems of interest have few bulk points and mostly contain points on a surface. For these systems, simply ignoring all points on the boundary is not an appropriate option, yet the full Delaunay tessellation contains unphysical connections. In such a case we can still use the alpha complex, which captures the topological neighborhood structure in a physically motivated way, but include the motifs on the boundary with large tetrahedrons removed in the motif distribution. The algorithms detailed here can run using these surface motifs, and we specify throughout when special cases of these algorithms arise due to such motifs. Whilst we have implemented this functionality, for consistency, we have not taken this approach for the systems here. Since the topological properties of the surface will typically differ from the bulk, for instance in the number of neighbors, by including surface information we are thus including global information about the ratio of surface to bulk points in the distribution which can be system size dependent. Instead, by only comparing bulk distributions throughout, we do not compare by system size but only by bulk topological properties.

D. Comparing structures

Given that we now characterize materials by the distribution of motifs, we need a way to compare these distributions. Methods such as Kullback-Leibler divergence or the Jensen-Shannon distance are able to compare distributions, however they are not aware of any structure on the space of motifs such as whether two motifs are similar in structure or not. This makes it difficult to compare distributions that have been undersampled [31], which is typically the case here. There is a natural metric on the space of motifs, which comes from the the idea of topological transitions. The Delaunay tessellation can only change through discrete topological transitions, and so the number of minimum number of flips to change one motif into another gives a notion of distance between motifs. These topological transitions, or flips, also gives a inherent graph structure, the flip graph, where two motifs are connected by an edge if they are one flip away from each other. The distance between motifs is then the minimum path length in this graph. Later, we introduce a spectral graph based distance, which can compare distributions in a way that is aware of the flip graph structure.

II. ALGORITHMIC IMPLEMENTATION

A. Storing motifs

Computationally, there is a need to store motifs in a concise representation allowing a set of motifs to be sorted into unique types, where two simplicial complexes are considered the same if some relabeling of the vertices makes them equal. In 2D this representation was the Weinberg vector [6,31], but this required a planar graph, and hence cannot work in 3D where we use the simplicial complex rather than the graph representation (which would not be planar in any case). This task corresponds to finding a canonical labeling for the vertices of our simplicial complex.

We start with the simplicial complex around a central vertex of some radius r. This consists of a number of d-simplices joined at their faces, where d is the dimension, and a vertex specified as the central vertex. We always label the central vertex 1. Next we choose an arbitrary d-simplex that contains 1, and label the other vertices $2, \ldots, d+1$, in some manner. We now construct a canonical labeling of the remaining vertices. Suppose we have labeled the first k vertices, and we need to choose which vertex to label $k+1$. The candidates for the $k+1^{th}$ vertex are all vertices that lie on a d-simplex that has the other d vertices labeled. Suppose that the other labeled vertices are $\{i_1, \ldots, i_d\}, \{j_1, \ldots, j_d\}, \ldots, \{l_1, \ldots, l_d\}$ for different candidate vertices. We choose the vertex to label based on the lexicographic ordering, i.e. if

$$\{i_1, \ldots, i_d\} < \{j_1, \ldots, j_d\} < \cdots < \{l_1, \ldots, l_d\}, \tag{S1}$$

then the $k+1^{th}$ vertex would be the one opposite to $\{i_1, \ldots, i_d\}$. Once all vertices are labeled, take the lexicographically ordered set of simplices as the identifier of the graph. The initial simplex was picked arbitrarily, as was the labeling of the vertices $2, \ldots, d+1$. To calculate the final canonical labeling, calculate the lexicographically ordered set of simplices for every possible initial labeling. Out of all of these simplices take the lexicographically first one, this is the canonical labeling of the graph, and two simplicial complexes will be isomorphic if and only if their canonically labeled set of simplices match. A worked example is shown in Fig. S4. In certain degenerate cases, these instructions are insufficient to fully label the simplicial complex and multiple possible labeling result from the same initial simpex labeling, see Fig. S4 for example and solution to this problem. In practice, for $r = 1$ in 3D, such a degenerate case has never been observed.

B. Computing the flip graph

The flip graph is the graph where every vertex is a motif, and two motifs are connected by an edge if they are one flip, or topological transition, away from each other in the Delaunay triangulation. Note that, unlike in 2D, a flip in 3D does not preserve the number of tetrahedrons, Fig. S5. We only consider topological transitions that arise through a continuous movement of vertices, not through inserting or deleting vertices. This results in there only being one type of flip in 3D [52]. The alpha complex will change through flips similarly, but can also change when the circumsphere radius of a tetrahedron becomes too large, and that tetrahedron is no longer in the alpha complex. When including surface motifs, we will consider this tetrahedron removal step as a type of flip.

In order to compare distributions over the flip graph, we must calculate the flip graph numerically. To do so, we make use of the fact that a flip either increases or decreases the number of tetrahedrons by one. For each observed motif, we calculate all motifs that can be reached by a flip that decreases the number of tetrahedrons, which will account for every possible flip. We note that by flipping in this direction, the distance of any vertex to the central vertex can only decrease, and hence the vertices of the post-flip local simplicial complex will be a subset of the pre-flip local simplicial complex. Whilst the only allowed operation is a flip, how this affects the local simplicial complex depends on which vertices affected by the flip are elements of the local simplicial complex. This results in a number of different cases.

Case 1: All vertices and simplices are in the initial simplicial complex. There are then two subcases, case 1A, Fig. S5, where all the post-flip vertices are in the simplicial complex, and case 1B, Fig. S5, where only 4 vertices are still in the post flip simplicial complex.

Case 2: Not all vertices are in the pre-flip simplicial complex. Case 2A, Fig. S5, has 4 vertices and 1 simplex in the pre-flip simplicial complex. Case 2B, Fig. S5, has 4 vertices and 0 simplices in the pre-flip simplicial complex. If a pre-flip simplicial complex had fewer than 4 vertices in it, it would not be affected by the flip.

Case 3: This is the special case that we only consider when including surface motifs. In this case a tetrahedron in the simplicial complex is removed. This occurs when the circumradius has become too large, but note that when we compute the flip graph, we never actually calculate the circumradius. We simply know that such an operation is possible.

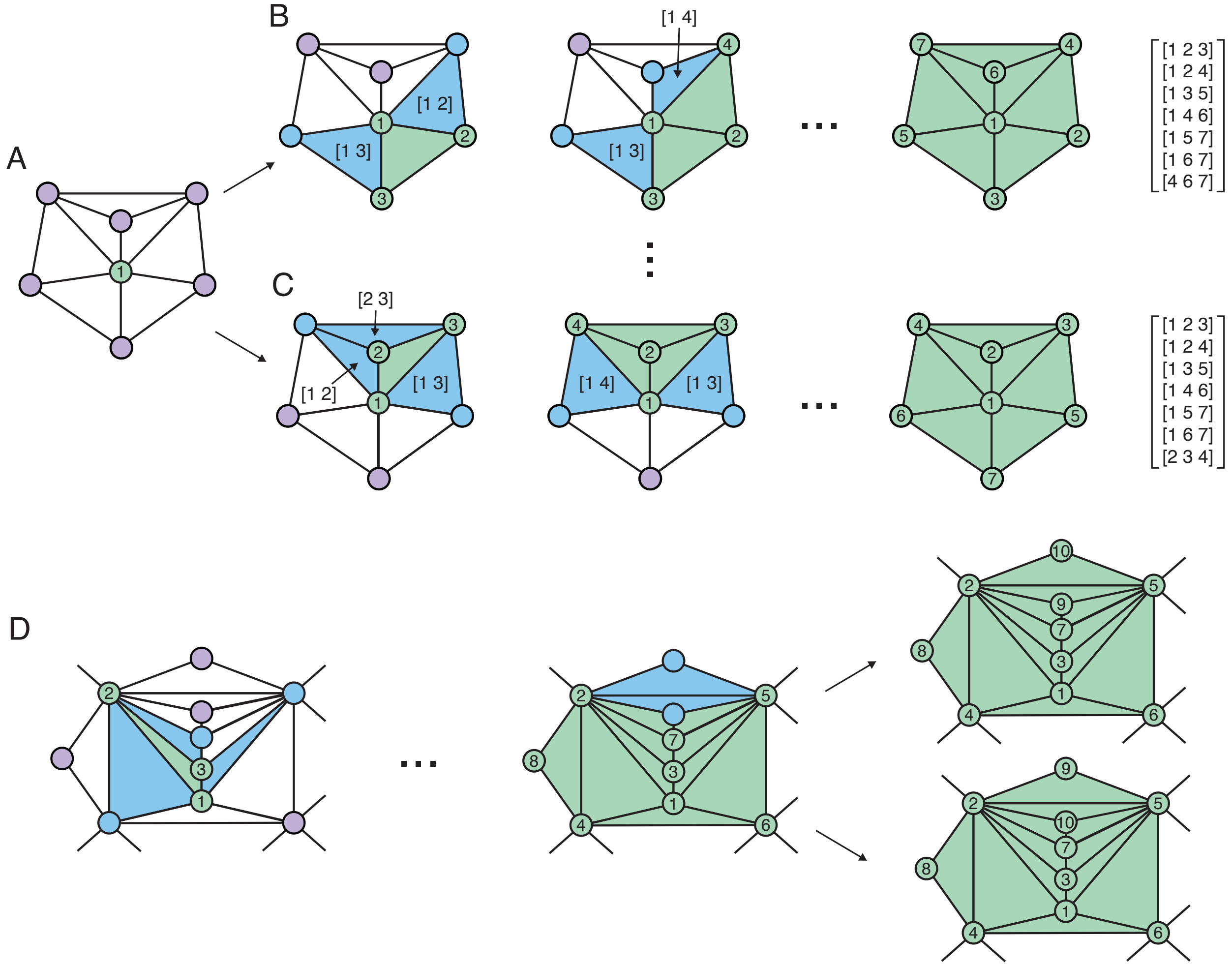

FIG. S4. A worked example of canonically labeling a motif, and an example of the degenerate case. Vertices and simplices that are green have been labeled, those in blue could be labeled in the next step, and purple is to be labeled. (A) A 2D motif with the central vertex labeled as vertex 1. (B) A simplex adjoining 1 is chosen and labeled. There are now two vertices which could be labeled next with two corresponding simplices. Each of these simplices has two vertices labeled already, which are (1 3), and (1 2). Since, lexicographically, (1 2) < (1 3), we label the 4th vertex as the one completing the simplex (1 2 4). Next, there are two vertices and corresponding simplices which could be labeled. As (1 3) < (1 4), the 5th vertex is the one which completes the simplex (1 3 5). This process continues until the motif is fully labeled, and the corresponding lexicographic ordered set of simplices is shown (right). (C) The procedure is repeated but with a different choice of initial labeling. The resulting labeling is different than (B), and results in a different set of simplices. The choice of labeling in (C) results in the minimum lexicographically ordered set of simplices, so this set of simplices serves as a topological identifier of this motif. (D) Part of a motif with an initial simplex labelled, which will result in a degenerate case. After several steps of the algorithm the next simplex to be completed should be (2 5 9), but there are two choices for vertex 9. This degeneracy is resolved by taking both options and choosing at the end the minimum lexicographically ordered set of simplices.

For every unique motif we calculate all motifs that are accessible by one flip that decreases the number of total simplices (Cases 1 and 2). In addition we calculate all motifs that can be reached by the reverse of flip 2A, in order to have a more connected flip graph, noting that this reverse flip can't increase the number of vertices in the local motif. If we are including surface motifs, then we also calculate motifs that are accessible through Case 3. From this newly enlarged set of motifs, we calculate which edges exist by taking all possible flips for every motif. Typically this results in a flip graph with over 95% of the original motifs in the largest connected component. If this flip graph proves too large for practical computations, we reduce it in size by calculating the page rank of each vertex, a measure of graph centrality. If the page rank of a motif is below some threshold, and that motif is not one of the original observed motifs, we remove it. By adjusting this threshold, one can typically reduce the graph size by a factor of 5 or so, with over 90% of original motifs still in the largest connected component of the reduced graph.

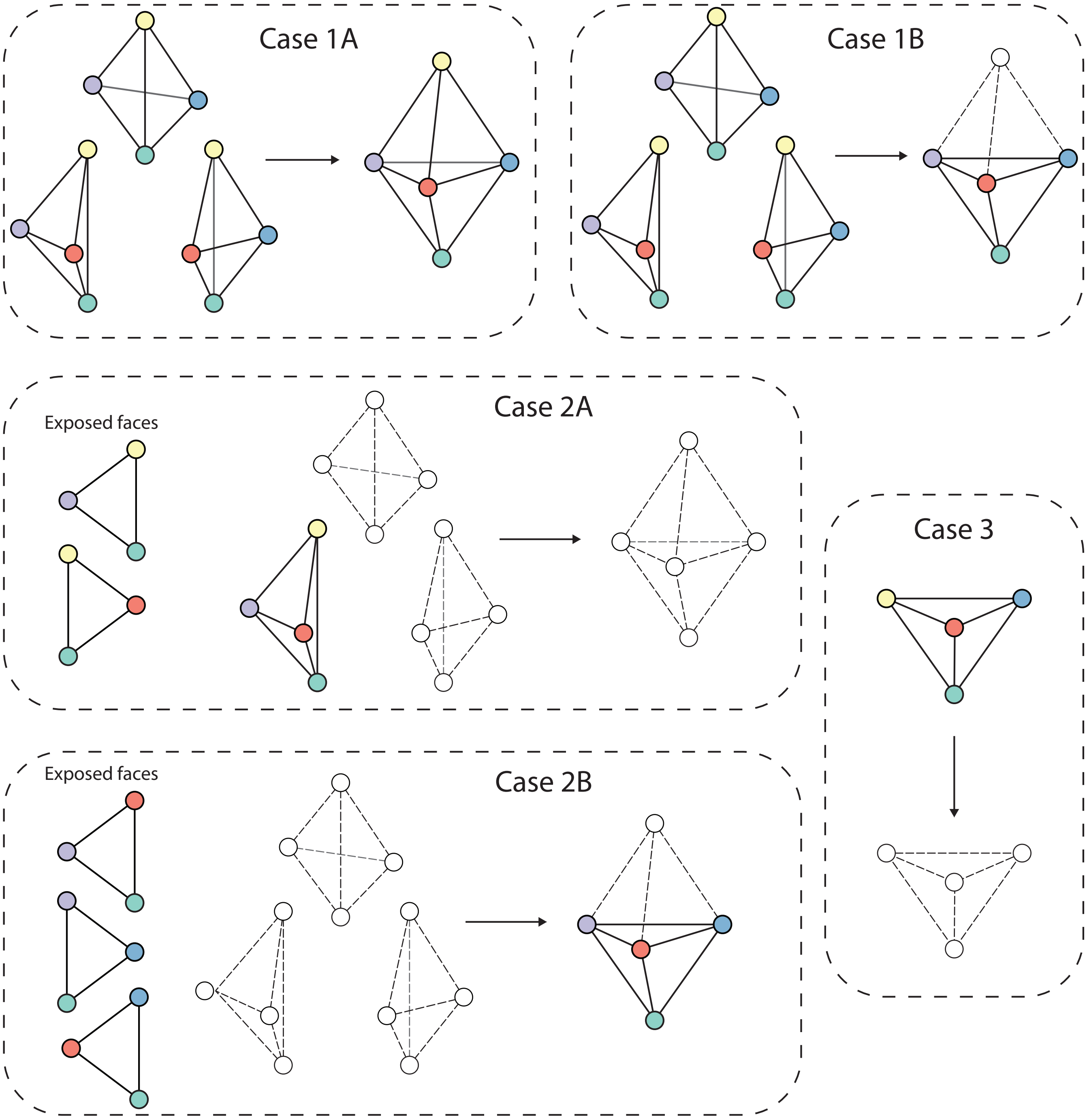

FIG. S5. Possible cases showing how a flip alters a motif, shown in the direction of reducing the number of simplices. **Case 1A**: All post-flip vertices are also in the simplicial complex. This is just the full flip, which we note does not preserve the number of tetrahedrons. **Case 1B**: Post-flip, a vertex (yellow) is no longer in the simplicial complex. **Case 2A**: 4 vertices and 1 simplex are in the pre-flip simplicial complex, but no simplices are in the post-flip simplicial complex. This can only happen to a local simplicial complex if certain faces are exposed as shown. **Case 2B**: 4 vertices and 0 simplices are in the pre-flip simplicial complex, and 1 simplex is in the post-flip simplicial complex. This can only happen to a local simplicial complex if certain faces are exposed as shown. **Case 3**: a simplex starts in the pre-flip simplicial complex, but is not in the post-flip simplicial complex. This case only occurs when we are including surface motifs.

III. CHOICE OF DISTANCE

A. Relaxing the earth mover's distance

Recall that given material A and material B, computing local motifs yields probability distributions ρ_A, and ρ_B over the discrete set of possible motifs. We have the additional structure of the flip graph, giving us a metric on the space of motifs, and we would like our comparison of ρ_A and ρ_B to be aware of this structure. One such comparison that is graph aware, is to use the Wasserstein or earth mover's distance to give a notion of distance between materials [31]. Computing the distance numerically is equivalent to solving a minimum cost flow problem.

Specifically, consider the flip graph to be directed with edges oriented arbitrarily. Then, let J_e be the flow along the eth directed edge. We also define the incidence matrix

$$D_{ev} = \begin{cases} 1 \text{ if } \exists\, w,\ e = (v,w), \\ -1 \text{ if } \exists\, w,\ e = (w,v), \\ 0 \text{ else.} \end{cases} \tag{S2}$$

The distance phrased as a minimum cost flow problem is then

$$d_{\text{TEM}}(A,B) = \min ||J||_1 \quad \text{subject to } D^T J = \rho_A - \rho_B. \tag{S3}$$

While there is nothing preventing us from using this distance, it becomes extremely expensive to compute for many of the datasets used here. In particular, the size of the flip graph in 3D perhaps a factor of 5-10 times larger than in 2D, making the minimum cost flow problem far more expensive. We have some discretion in how large the flip graph is, for instance by only including motifs that have been observed more than n times for some n, or by setting the page-rank threshold to be higher, and hence restricting the graph size. However, we can get a huge reduction in computational time by changing the distance we use, whilst not compromising on our goal of having a distance that is aware of the flip graph structure. We introduce this distance now.

Following Ref. [53], we start by rewriting our expression for the earth mover's distance by using a Helmholtz-like decompositon of the flow as

$$J = J_0 + Df, \tag{S4}$$

where $D^T J_0 = 0$ and f is arbitrary, the decomposition follows from rank nullity. The constraint then becomes

$$D^T D f = \rho_A - \rho_B, \tag{S5}$$

in which we recognise $L = D^\top D$ as the discrete graph laplacian. Taking the pseudo-inverse, $f = L^+(\rho_A - \rho_B)$, the minimization problem is

$$d_{\text{TEM}}(A,B) = \min ||J_0 + DL^+(\rho_A - \rho_B)||_1 \text{ subject to } D^T J_0 = 0. \tag{S6}$$

Since J_0 is some element in the kernel $D^T x = 0$, we can write $J_0 = \sum_{i=1}^N a_i K_i$, where the K_i's form a basis for the kernel and the a_i's are coefficients. Formally, the minimization is

$$d_{\text{TEM}}(A,B) = \min_{a_i} ||\sum_{i=1}^{N} a_i K_i + DL^+(\rho_A - \rho_B)||_1, \tag{S7}$$

which now has no constraints. It is also possible to define a family of distances using a restricted sum on the kernel vectors [53],

$$d_M(A,B) = \min_{a_i} ||\sum_{i=1}^{M} a_i K_i + DL^+(\rho_A - \rho_B)||_1, \tag{S8}$$

for $0 \leq M < N$. For graphs that come from the discretization of a smooth manifold, one can observe spectral convergence and thus approximate $d_{TW} \approx d_M$ for $M \ll N$ [53]. The flip graph does not arise from a smooth manifold, and so we observe only linear convergence. A large number of kernel vectors would therefore be needed for a good approximation to the earth mover's distance, saving minimal computational time. We consider instead the distance defined by taking $M = 0$, hence,

$$d_{\text{TDD}}(A,B) = ||DL^+(\rho_A - \rho_B)||_1. \tag{S9}$$

This defines a distance that can be calculated simply by solving a linear system of equations. While there is no guarantee that $d_{TDD} \approx d_{TEM}$, we do not need this property since d_{TDD} is a well defined distance metric on its own that is aware of the graph structure. Moreover, we show that, like the earth mover's distance, it has a natural physical interpretation.

The diffusion equation for a scalar field ϕ over a graph with sources and sinks of strength $\rho_A - \rho_B$, is

$$\frac{\mathrm{d}\phi}{\mathrm{d}t} + L\phi = \rho_A - \rho_B, \tag{S10}$$

with diffusion constant set to 1. The steady state of this equation, has

$$\phi = L^{+}(\rho_A - \rho_B) + const., \tag{S11}$$

and the net flow rate is given by

$$J = D\phi. \tag{S12}$$

Therefore the L_1 cost of the flow is

$$d_{\mathrm{TDD}}(A,B) = ||D\phi||_1 = ||DL^{+}(\rho_A - \rho_B)||_1. \tag{S13}$$

For optimal transport, the natural interpretation was that it was the minimum number of flips needed to make distribution A look like distribution B. This could also be interpreted physically, as the energetic distance between distributions when each flip is associated with crossing an energy barrier as with epithelial cells. The distance d_{TDD} also has a natural interpretation; it is the number of flips needed to make distribution A look like distribution B, but without any control over which flip occurs. The probability mass undergoes diffusion on the graph with sources and sinks, and the rate of flipping corresponds to the L_1 norm of the flow.

In many ways the diffusive distance $d_{\mathrm{TDD}}(A,B)$ is more physically appealing than the earth mover's distance, $d_{\mathrm{TEM}}(A,B)$, random flips are more physically realizable than a targeted minimization over all possible flows. The computation is orders of magnitude faster and involves solving a sparse linear system, for which optimized iterative solvers exist. In any case, as long as the distance used is aware of the underlying metric on motifs, the key conceptual ideas of the method are utilized.

Whilst the earth mover's distance may be too expensive to use for certain computations done here, we were able to compute the pairwise earth mover's distance between all pairs of biofilm experiments, enabling us to compare directly to the diffusion distance. We find that, whilst both distances result in a similar embedding, there is actually more structure in the diffusion distance embedding, Fig. S6. From now on, we therefore use the diffusion distance.

B. Comparison to Bottleneck distance

Whilst the framework introduced here takes a topological approach to analyze data, our approach is distinct from the field of topological data analysis (TDA). As originally developed, TDA seeks to understand the "shape" of a high dimensional data manifold by characterizing it topologically [1]. Typically, the starting point is the Vietoris-Rips complex, a topological object somewhat related to the Voronoi diagram, but containing more detailed information, and from this further features are computed, such as the number of n-dimensional "holes" [1]. These resulting features can be summarized using the persistence diagram which, ultimately, represents key topological features as a set of points in $\mathbb{R}^2$ [1]. Two objects, say different biofilms, can then be compared by comparing their persistence diagrams. a A common way to compare persistence diagrams is to use the bottleneck distance, which, as each persistence diagram is a collection of points in $\mathbb{R}^2$, is essentially the earth mover's distance between these two collections of points, but accounting for an unequal number of points [54].

We use the computational framework of Ref. [55], to compute the pairwise bottleneck distance between different biofilms using their one dimensional persistence diagram. From this distance matrix, we embedded the points into Euclidean space with MDS. This embedding does not reveal any of the structure of the data, Fig. S6C, and whilst the *E. coli* form a somewhat distinct cluster, different species are all intermixed. Moreover, this computation was orders of magnitude slower than computing the diffusion distance. This is not surprising as the bottleneck distance is not physically motivated for the 3D systems studied here. Specifically, whilst understanding the 'shape" of a data manifold is a key challenge for high dimensional data, for the 3D data we work with, the local structure is more important physically than larger scale features such as holes.

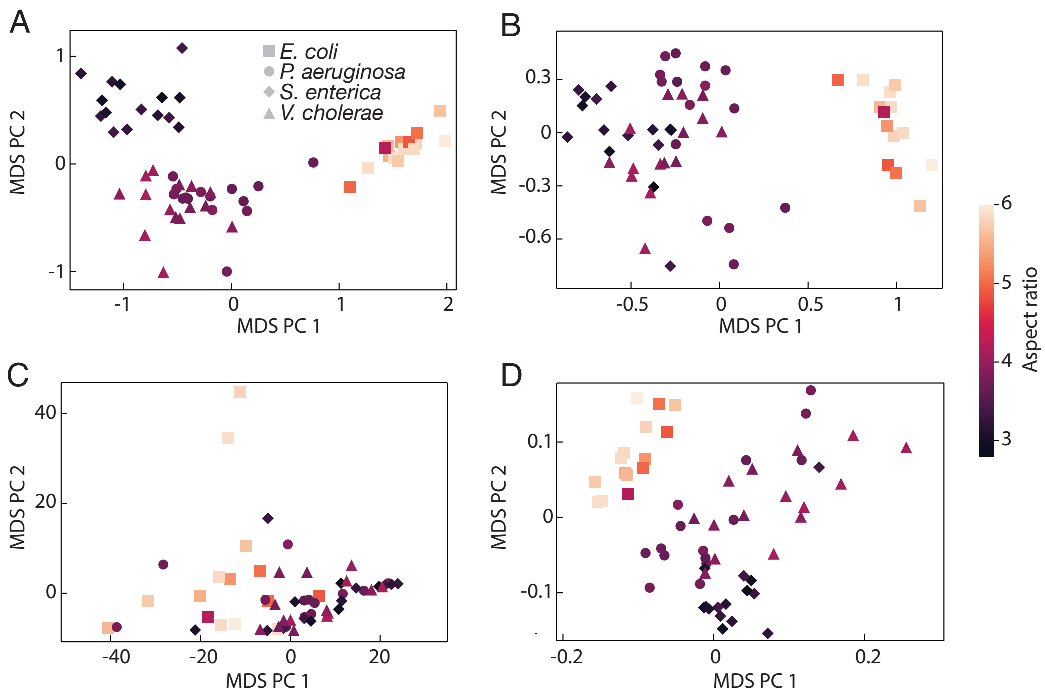

FIG. S6. Comparison of the diffusion based distance d_{TDD}, the earth mover's distance d_{TEM}, and the bottleneck distance, d_{BN}, as applied to bacterial biofilm data. (A) 2D embedding of the diffusion distance colored by average cellular aspect ratio, as appears in Fig. 1D. (B) Embedding of the earth mover's distance is broadly similar to the diffusion distance embedding, but does not separate *S. enterica* as clearly from *P. aeruginosa* and *V. cholerae* biofilms. (C) The Bottleneck distance embedding shows no discernible structure and fails to separate out different species of biofilm. (D) Embedding based on the Jensen-Shannon (JS) distance is slightly worse than the earth mover's distance and is unable to separate fully *S. enterica* from the other species, but outperforms the bottleneck distance.

C. Comparison to Jensen-Shannon distance

A widely used class of distances compare distributions based on quantities related to their relative entropies, such as the the Kullback-Leibler divergence or the Jensen-Shannon (JS) distance. Such distances arise naturally in information theory, but are unaware of any metric, in particular they do not use the graph structure on the space of motifs. The JS distance between discrete distributions p and q is

$$d_{JS}(p,q)^2 = \frac{1}{2}\sum_i p_i \log\frac{p_i}{z_i} + q_i \log\frac{q_i}{z_i}, \qquad z_i = \frac{1}{2}(p_i + q_i), \tag{S14}$$

where we note that this distance only uses frequencies of states and not any structural information about the underlying space. In 2D, we previously found that JS distances performed worse than optimal transport, particularly for subsampled distributions [31]. Here, we find that the TDD outperforms JS, Fig. S6D, although JS still performs substantially better than the TDA bottleneck distance whilst requiring orders of magnitude less computational cost. We also repeated the biofilm bootstrapping calculation, that will be described in Section V, using the JS distance. We found that whilst the TDD can distinguish *V. cholarea* and *P. aeruginosa* at $p < 0.01$, the JS distance can not, although it can distinguish all other pairs at $p < 0.01$. The bootstrap calculation was too expensive to perform with the earth mover's or bottleneck distances.

D. Behavior under subsampling

With the large number of motifs observed for any given system, $O(10^6)$ in total, including a large number that were only observed once, we conclude that we are typically undersampling the motif distribution. This could pose an issue were we using, say, a Jensen-Shannon distance, but the fact that our distance makes use of the flip graph structure of motifs allows us to be robust to undersampling. As an analogy, consider numerically drawing two samples

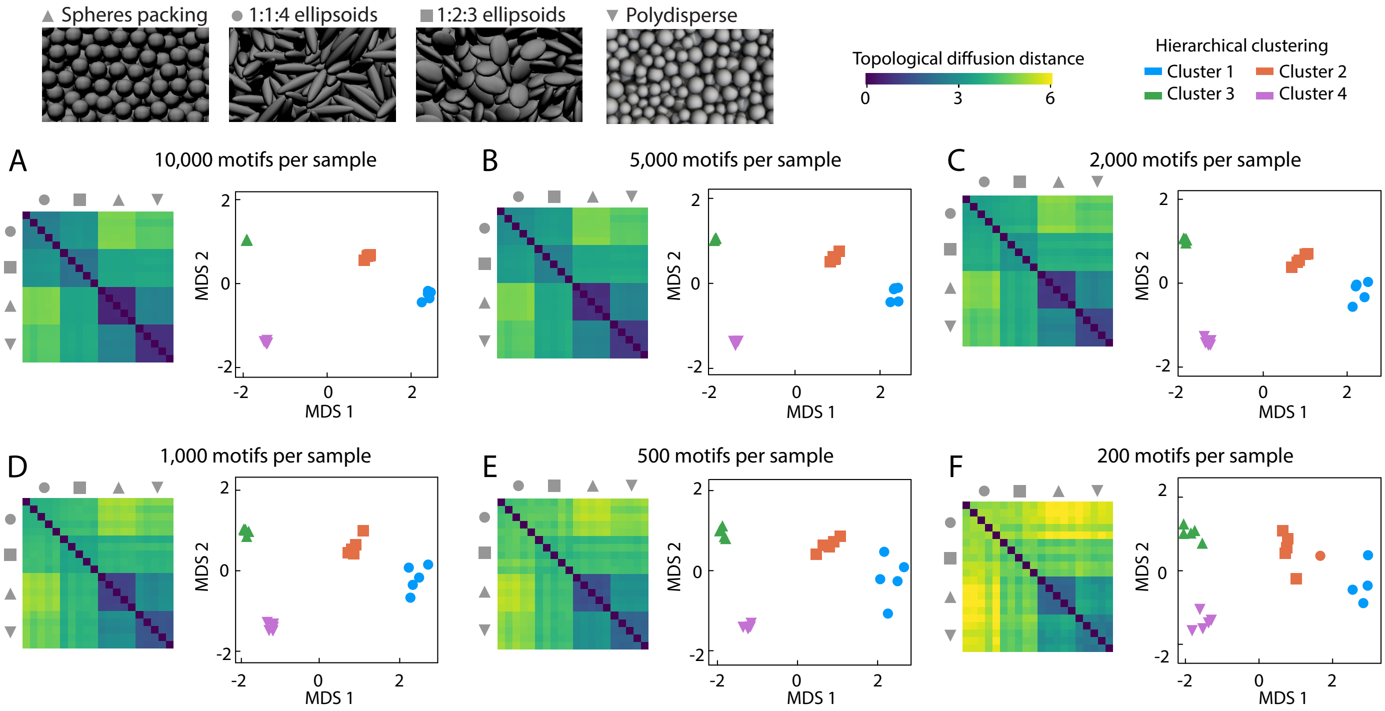

FIG. S7. Topological metric can distinguish between different particle packings using as few as 200 motifs. Four different types of particle packings were simulated, a regular sphere packing, 1:1:4 aspect ratio ellipsoids, 1:2:3 aspect ratio ellipsoids, and a polydisperse packing of spheres (for details see Sec. VI). (A) The pairwise distance matrix (left) was computed for 5 simulations for each packing type (20 simulations total, each simulation contains 10,000 particles), along with the resulting MDS embedding (right). The distance matrix shows a clear block structure, distinguishing the different cell types, and a hierarchical clustering of the distance matrix recovers back the true clusters (color of MDS embedding). (B-D) Subsampling the motif distribution so it contains fewer motifs per sample does not substantially change the distance matrix structure, although some of the finer details are degraded at 1000 or 500 motifs per sample, such as the difference in distance between two 1:1:4 ellipsoid packings and a 1:1:4 vs 1:2:3. Nonetheless, the hierarchical clustering still recovers the correct 4 clusters corresponding to packing type. (E) Even at 200 motifs per sample, the distance matrix retains clear structure. A hierarchical clustering places one of the 1:1:4 ellipsoids in the 1:2:3 ellipsoid cluster, but otherwise correctly clusters all other points.

from a normal distribution $\mathcal{N}(0,1)$ and two samples from a normal distribution $\mathcal{N}(1000,1)$, resulting in 4 empirical distributions each containing a single number. All 4 numbers will come from the discrete set of 64 bit floats, and (almost certainly) every number will be different. Without knowledge of the metric structure of $\mathbb{R}$, we have hopelessly undersampled and each empirical distribution is equally distinct from each other. However, knowledge of the structure of $\mathbb{R}$ allows us to recognize that the two numbers near zero are more similar to each other than to the two numbers near 1000. Hence, using a distance which is aware of the metric structure generically should be more robust to undersampling than one that is not.

To confirm this robustness in the context of generic disordered structures, we take 4 types of particle packings; a regular sphere packing, 1:1:4 aspect ratio ellipsoids, 1:2:3 aspect ratio ellipsoids, and a polydisperse packing of spheres (for details see Sec. VI). Using a simulation of 10,000 particles, different types of packings can be clearly distinguished from each other, with little variation between simulations of the same packing type, Fig. S7A. Subsampling these simulations so they contain fewer particles begins to degrade the structure of the distance matrix, but the resulting MDS embedding recovers the different types of packing even with just 200 motifs per sample, Fig. S7F. A hierarchical clustering with 200 motifs per sample only misclassifies a single sample, and gets all samples correct for 500 motifs per sample and above, Fig. S7.

IV. MENGER CURVATURE

Our topological diffusion distance creates a metric space where points are distributions over the space of motifs. Remarkably, the field of distance geometry allows us to build geometric concepts from such an abstract metric space

alone [38]. For instance, a well known and arguably the earliest result in distance geometry is Hero's formula for the area of a triangle; allowing the computation of the area from only the distances between points. Later work by Arthur Cayley and Karl Menger established conditions for an abstract (semi-)metric space to be equivalent to $\mathbb{R}^n$ for some n [38]. Here, we introduce a geometric notion of curvature along a 1D path in topological space, which could be extended to define curvature for a surface or general manifold.

Consider a topological distribution that changes with some parameter, for instance time, ρ_t. We would expect the MDS embedding of such a process to recover the temporal ordering, as indeed we find in Fig. 2D (main text) for zebrafish development. However, the embedding may take a highly curved path, as was seen in Fig. 2D, or follow a straight line, as was found in two dimensions for the development of a fly wing [31]. In Euclidean space, a straight line is the optimal way to move between two points, whereas a curved path is longer. This gives us an intuition in our topological space that a system which takes a straight path acts to minimize the number of topological flips, whereas a system with a curved path performs more flips, and hence more rearranging, than is necessary. In making this intuition precise, we wish to avoid the distorting effect that a low dimensional embedding may have on a trajectory, and so we want to have a notion of how curved the path is at a point, independent of the embedding.

To start, we consider the notion of a straight line, or geodesic, under the d_{TDD} distance. A path ρ_t between ρ_0 and ρ_1 is a geodesic if

$$\sum_{i=0}^{n} d_{TDD}(\rho_{t_i}, \rho_{t_{i-1}}) = d_{TDD}(\rho_0, \rho_1), \tag{S15}$$

for all $0 = t_0 < \cdots < t_n = 1$. Not all paths are geodesics, but as with the space of Wasserstein 1 (W_1) or earth mover's distances [56], geodesics are not unique in the space of d_{TDD}.

For instance, an example of an optimal path would be $\rho_t = \rho_0 + t(\rho_1 - \rho_0)$, where given any finite $0 = t_0 < t_1 < \cdots < t_n = 1$,

$$\sum_{i=1}^{n} d_{TDD}(\rho_{t_i}, \rho_{t_{i-1}}) = \sum_{i=1}^{n} ||DL^{\dagger}(\rho_{t_i} - \rho_{t_{i-1}})||_1 = \sum_{i=1}^{n} ||DL^{\dagger}(t_i - t_{i-1})(\rho_1 - \rho_0)||_1 = d_{TDD}(\rho_0, \rho_1). \tag{S16}$$

However, this path corresponds to phase separated growth, at time t, a fraction t of the system is in the ρ_1 phase, and a fraction $1 - t$ is in the ρ_2 phase. To identify the most physical continuous path between ρ_0 and ρ_1 we will need additional structure beyond the d_{TDD} distance.

Following our previous work in 2D, and building on the work of Ref. [56], we can find the most natural geodesic by choosing the path between ρ_0 and ρ_1 that additionally minimizes a dissipation like term.

$$\begin{aligned} &\inf_{J(t,e),p(v,t)\geq 0} \int_0^1 \sum_{e=(u,v)} \frac{J(t,e)^2}{2} \left(\frac{1}{p(t,u)} + \frac{1}{p(t,v)} \right) \mathrm{d}t \\ &\text{such that,} \quad \sum_u p(t,u) = 1, \ p(0,v) = \rho_0(v), \ p(1,v) = \rho_1(v), \\ &\frac{\mathrm{d}}{\mathrm{d}t} p = D^{\top} J, \qquad \sum_{i=1}^{n+1} d_{TDD}(p(t_i, \cdot), p(t_{i+1}, \cdot)) = d_{TDD}(\rho_0, \rho_1), \end{aligned} \tag{S17}$$

where we are minimizing the square of a current $J(t, e)$ along an edge e divided by probability mass, which has the interpretation of a squared velocity multiplied by a probability mass; a dissipation like term [56]. The resulting infimum $p(t, \cdot)$ gives us the unique dissipation minimizing geodesic path. The final condition ensures that any path that is taken is still a geodesic under the d_{TDD} metric, and we will prove later that condition is automatically enforced by the local minimization and does not need to be additionally imposed.

The interpretation of the (unique) infimum, J and p, is that they describe a unique geodesic between ρ_0 and ρ_1. One can interpret this geodesic as the minimum dissipation path to move mass from ρ_0 to ρ_1, and it results in mass being locally transported across the flip graph, rather than being moved discontinuously [56].

Instead of choosing a special path out of many geodesics of d_{TDD}, these geodesics arise naturally from an alternate

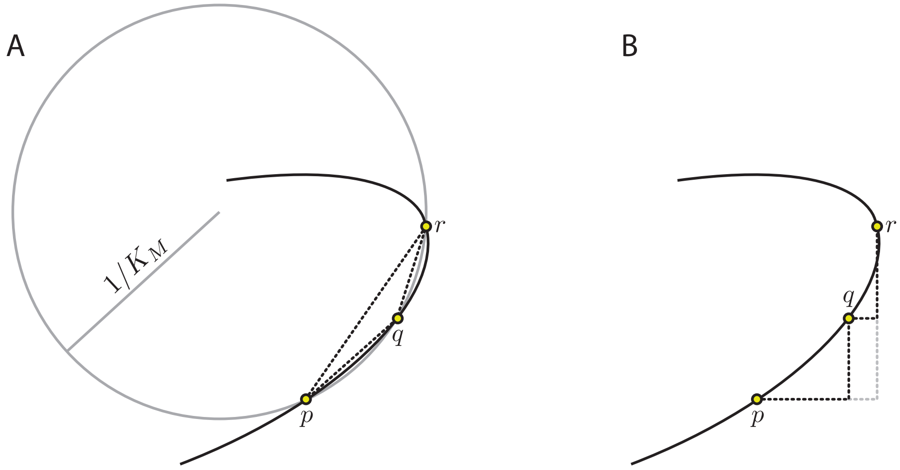

FIG. S8. Menger curvature for a plane curve with three points p, q, r. (A) The points p, q, r on some curve form a triangle in the plane, with corresponding circumcircle shown in gray. The radius of this circumcircle can be found by only knowing the pairwise distances between the points, using a well known formula, and Menger curvature $K_M(p,q,r)$ is defined to be the reciprocal of this radius. Since this formula, Eq. (S19), only uses pairwise distances, it can be extended to give a concept of curvature in any metric space. (B) With the L_1 metric in the plane, the points p, q, r lie on a straight line, as the path going through all three (black dashed line) is just as long as the direct path between p and r (gray dashed line). In this case, the Menger curvature would be zero.

distance $\bar{W}$ between two distributions [56], namely

$$[\bar{W}(\rho_0,\rho_1)]^2 = \inf_{J(t,e),p(v,t)\geq 0} \int_0^1 \sum_{e=(u,v)} \frac{J(t,e)^2}{2}\left(\frac{1}{p(t,u)}+\frac{1}{p(t,v)}\right)\mathrm{d}t \tag{S18}$$

$$\text{such that,}\quad \sum_u p(t,u)=1,\ p(0,v)=\rho_0(v),\ p(1,v)=\rho_1(v),$$

$$\frac{\mathrm{d}}{\mathrm{d}t}p = D^\top J.$$

Computing the $\bar{W}$ distance requires solving a second-order conic system, which is even more expensive than the W_1 computation, and making it impractical for the size of systems we work with. However, we will show that we can use the properties of this distance to define a notion of local curvature, relating how close an observed path comes to the dissipation minimizing geodesic, without ever needing to explicitly calculate $\bar{W}$.

Generally speaking, consider that we observe a path ρ_t, in some space where a unique geodesic arises from some metric d. We wish to to quantify how close our path is to being a geodesic. To do so, we draw on motivation from Euclidean geometry, even though the following definition applies for any metric space. We define the Menger curvature [57] of points p,q,r as follows,

$$K_M(p,q,r) = \frac{\sqrt{[pq+qr+rp][-pq+qr+rp][pq-qr+rp][pq+qr-rp]}}{pq\cdot qr\cdot rp} \tag{S19}$$

for a general metric $d : X\times X\to\mathbb{R}$, and $pq := d(p,q)$, etc.. This is also the inverse of the radius of curvature of a triangle with side lengths $d(p,q)$, $d(q,r)$, and $d(r,p)$, see Fig. S8. The curvature quantifies the extent to which, locally, the path does not take the shortest path under the metric d.

To locally define a curvature, for our curve ρ_t, we would like to take $K_M(\rho_t,\rho_{t+\Delta t},\rho_{t-\Delta t})$ in the limit $\Delta t\to 0$ and this the curvature of the path at ρ_t. For case of the L_1 metric in the plane, this will result in almost all points having zero curvature, Fig. S8B (a measure zero set may have a diverging curvature). Similarly, with d_{TDD} based on an underlying L_1 metric, the curvature K_M will be almost everywhere zero, as locally many paths will be geodesics. However, using the $\bar{W}$ distance, which gives rise to a unique geodesic, will generally result in a non-vanishing curvature. We therefore seek to use $\bar{W}$ to compute the curvature along a curve, and specifically we are using this curvature as a measure of how much a given path differs from the dissipation minimizing path under d_{TDD} which locally is governed by the metric $\bar{W}$. As we only need to compute $\bar{W}$ between asymptotically similar distributions ρ_t,$\rho_{t+\Delta t}$,$\rho_{t-\Delta t}$, we

need not solve a second order conic system, and the computational cost is only slightly more expensive than computing the d_{TDD} distance.

A. Curvature perturbation problem

Formally, we seek an asymptotic solution of the $\bar{W}$ distance between distributions $p = p_0 + \epsilon p_1 + \epsilon^2 p_2 + \dots$, and $r = p_0 + \epsilon r_1 + \epsilon^2 r_2 + \dots$, where ϵ is small, and by construction p and r converge to the same distribution in the $\epsilon \to 0$ limit. We have an expansion of the form $J = \epsilon J_1 + \epsilon^2 J_2 + \dots$, and $q = p_0 + \epsilon q_1 + \epsilon^2 q_2 + \dots$, and plugging these into Eq. (S18) gets the following hierarchy,

$$\begin{aligned}\bar{W}^2 =& \epsilon^2 I_0(J_1, q_1, p_0, p_1, r_1) + \epsilon^3 I_1(J_1, J_2, q_1, q_2, p_0, p_1, p_2, r_1, r_2) \\ &+ \epsilon^4 I_2(J_1, J_2, J_3, q_1, q_2, q_3, p_0, p_1, p_2, p_3, r_1, r_2, r_3) + \dots,\end{aligned} \tag{S20}$$

where each I_j is a minimization problem, and higher order I_j's depend on variables that were fully or partially determined by lower order ones. We will need to solve up to I_2, and also note here that

$$\bar{W} = \epsilon\sqrt{I_0} + \frac{\epsilon^2}{2}\frac{I_1}{\sqrt{I_0}} + \epsilon^2\left[\frac{1}{2}\frac{I_2}{\sqrt{I_0}} - \frac{1}{8}\frac{I_1^2}{I_0^{3/2}}\right] + \dots, \tag{S21}$$

which we will make use of when computing the curvature later.

1. Zeroth order

At the lowest order, the problem we are trying to solve becomes

$$\begin{aligned} I_0 = \inf_{J_1, q_1} \int_0^t \sum_{e=(u,v)} \frac{J_1(t,e)^2}{2}\left(\frac{1}{p_0(u)} + \frac{1}{p_0(v)}\right) \mathrm{d}t \\ \text{such that,} \quad q_1|_{t=0} = p_1,\ q_1|_{t=1} = r_1, \\ D^\top J_1 = \dot{q}_1,\end{aligned} \tag{S22}$$

where no requirement that q_1 must be non-negative exists. Now that we are free from all positivity constraints, we can find the minimum by using Lagrange multipliers

$$\mathcal{L} = \int_0^t \sum_{e=(u,v)} \frac{J_1(t,e)^2}{2}\left(\frac{1}{p_0(u)} + \frac{1}{p_0(v)}\right) + \sum_w \lambda_0(t,w)\left(\sum_{e=(u,v)} D_{ew} J_1(t,e) - \dot{q}_1(t,w)\right) \mathrm{d}t, \tag{S23}$$

where a minimizing solution satisfies

$$\frac{\delta\mathcal{L}}{\delta J_1} = J_1(t,e)\left(\frac{1}{p_0(u)} + \frac{1}{p_0(v)}\right) + \sum_w D_{ew}\lambda_0(t,w) = 0, \tag{S24}$$

$$\frac{\delta\mathcal{L}}{\delta q_1} = \dot{\lambda}_0(t,w) = 0. \tag{S25}$$

From this we can immediately identify that the Lagrange multiplier λ_0 does not depend on time, thus neither does J_1, and so q_1 is linear in time, and moreover we can deduce q_1 from the initial and final conditions. We therefore have that

$$q_1 = (1-t)p_1 + t r_1, \tag{S26}$$

$$J_1(e)\left(\frac{1}{p_0(u)} + \frac{1}{p_0(v)}\right) + \sum_w D_{ew}\lambda_0(w) = 0, \tag{S27}$$

$$\sum_{e=(u,v)} D_{ew} J_1(e) = r_1(w) - p_1(w). \tag{S28}$$

From now on, understanding $D = (D_{ev})$ to only have indices that run over edges $e = (u, v), u < v$, and defining

$$\Lambda_{ee'} = \delta_{ee'} \left(\frac{1}{p_0(u)} + \frac{1}{p_0(v)} \right), \tag{S29}$$

we have that

$$\Lambda J_1 + D\lambda_0 = 0 \implies D^\top J_1 + D^\top \Lambda^{-1} D\lambda_0 = 0 \implies L\lambda_0 = -(r_1 - p_1), \tag{S30}$$

where $L = D^\top \Lambda D$, and whilst the solution of $L\lambda_0 = p_1 - r_1$ is not unique, it will always lead to the same J_1, which is unique. Overall,

$$I_0 = -\lambda_0^\top (r_1 - p_1), \quad \text{where } L\lambda_0 = -(r_1 - p_1) \tag{S31}$$

2. First order

At the next order, we are trying to solve

$$I_1 = \inf_{J_2^i, q_2^i} \int_0^1 \sum_{e=(u,v)} J_2(t,e) J_1(e) \left(\frac{1}{p_0(u)} + \frac{1}{p_0(v)} \right) + \frac{J_1(e)^2}{2} \left(-\frac{q_1(t,u)}{p_0(u)^2} - \frac{q_1(t,u)}{p_0(v)^2} \right) \mathrm{d}t \tag{S32}$$
$$\text{such that,} \quad q_2^0 = p_2,\ q_2^k = r_2, \quad D^\top J_2 = \dot{q}_2,$$

which in light of the known form of J_1, q_1 we can rewrite as

$$I_1 = \inf_{J_2, q_2} \sum_{e=(u,v)} \left[\int_0^1 J_2(t,e) \mathrm{d}t \right] \Lambda_{ee} J_1(e) - \sum_{e=(u,v)} \frac{J_1(e)^2}{4} \left(\frac{r_1(u) + p_1(u)}{p_0(u)^2} + \frac{r_1(v) + p_1(v)}{p_0(v)^2} \right) \tag{S33}$$
$$\text{such that}, D^\top \left[\int_0^1 J_2(t,e) \mathrm{d}t \right] = r_2 - p_2,$$

where we can rewrite part of the objective as

$$\sum_{e=(u,v)} \left[\int_0^1 J_2(t,e) \mathrm{d}t \right] \Lambda_{ee} J_1(e) = - \sum_{e=(u,v),w} \left[\int_0^1 J_2(t,e) \mathrm{d}t \right] D_{ew} \lambda_0(w) = - \sum_w (r_2(w) - p_2(w)) \lambda_0(w) \tag{S34}$$

so that in total

$$I_1 = - \sum_w (r_2(w) - p_2(w)) \lambda_0(w) - \sum_{e=(u,v)} \frac{J_1(e)^2}{4} \left(\frac{r_1(u) + p_1(u)}{p_0(u)^2} + \frac{r_1(v) + p_1(v)}{p_0(v)^2} \right) \tag{S35}$$

and at this level, we need not find J_2 nor q_2 explicitly.

3. Second order

At second order we are solving

$$I_2 = \inf_{J_2, q_2, J_3, q_3} \int_0^1 \sum_{e=(u,v)} \left[J_3(t,e) J_1(e) + \frac{J_2(t,e)^2}{2} \right] \left(\frac{1}{p_0(u)} + \frac{1}{p_0(v)} \right) + J_1(e) J_2(t,e) \left(-\frac{q_1(t,u)}{p_0(u)^2} - \frac{q_1(t,v)}{p_0(v)^2} \right) \tag{S36}$$
$$+ \frac{J_1(e)^2}{2} \left(\frac{q_1(t,u)^2/2 - p_0(u) q_2(t,u)}{p_0(u)^3} + \frac{q_1(t,v)^2/2 - p_0(v) q_2(t,v)}{p_0(v)^3} \right) \mathrm{d}t$$
$$\text{such that,}\ q_2|_{t=0} = p_2,\ q_2|_{t=1} = r_2,\ D^\top J_2 = \dot{q}_2,\ q_3|_{t=0} = p_3,\ q_3|_{t=1} = r_3,\ D^\top J_3 = \dot{q}_3.$$

Firstly, by the same logic as at first order, we know the term involving J_3 as

$$\sum_{e=(u,v)} \left[\int_0^1 J_3(t,e) \mathrm{d}t \right] \Lambda_{ee} J_1(e) = - \sum_w (r_3(w) - p_3(w)) \lambda_0(w) \tag{S37}$$

and moreover, we will see later that this term cancels in the curvature computation. We can also compute

$$\chi_3 = \int_0^1 \sum_{e=(u,v)} \frac{J_1(e)^2}{4}\left(\frac{q_1(t,u)^2}{p_0(u)^3} + \frac{q_1(t,v)^2}{p_0(v)^3}\right) \mathrm{d}t \tag{S38}$$

$$= \frac{J_1(e)^2}{12}\left(\frac{r_1(u)^2 + r_1(u)p_1(u) + p_1(u)^2}{p_0(u)^3} + \frac{r_1(u)^2 + r_1(u)p_1(u) + p_1(u)^2}{p_0(v)^3}\right).$$

We can write the remaining minimization problem with constraints as Lagrange multipliers as

$$\mathcal{L} = \int_0^1 \sum_{e=(u,v)} \frac{J_2(t,e)^2}{2}\left(\frac{1}{p_0(u)} + \frac{1}{p_0(v)}\right) + J_1(e)J_2(t,e)\left(-\frac{q_1(t,u)}{p_0(u)^2} - \frac{q_1(t,v)}{p_0(v)^2}\right) \tag{S39}$$

$$+ \frac{J_1(e)^2}{2}\left(\frac{-q_2(t,u)}{p_0(u)^2} + \frac{-q_2(t,v)}{p_0(v)^2}\right) + \sum_w \lambda_1(w)\left(\sum_e D_{ew}J_2(t,e) - \dot{q}_2(w)\right) \mathrm{d}t,$$

or

$$\mathcal{L} = \int_0^1 \frac{1}{2}J_2^\top \Lambda J_2 + J_1^\top \tilde{\Lambda} J_2 + \Gamma^\top q_2 + \lambda_1^\top \left(D^\top J_2 - \dot{q}_2\right) \mathrm{d}t, \tag{S40}$$

in concise matrix notation, with

$$\Gamma(u) = \sum_v \frac{J_1(e=(u,v))^2}{2p_0(u)^2} + \sum_v \frac{J_1(e=(v,u))^2}{2p_0(u)^2}, \tag{S41}$$

where the sum is taken over edges that exist without double counting. We find the following,

$$\frac{\delta\mathcal{L}}{\delta J_2} = \Lambda J_2 + \tilde{\Lambda} J_1 + D\lambda_1 = 0, \tag{S42}$$

$$\frac{\delta\mathcal{L}}{\delta q_2} = \Gamma + \dot{\lambda}_1 = 0, \tag{S43}$$

from which we deduce that J_2 is linear in time, and q_2 is quadratic. Calling

$$\tilde{\Lambda} J_1 = J_1(e)\left(-\frac{q_1(t,u)}{p_0(u)^2} - \frac{q_1(t,v)}{p_0(v)^2}\right) \tag{S44}$$

$$= J_1(e)\left(-\frac{(1-t)p_1(u) + tr_1(u)}{p_0(u)^2} - \frac{(1-t)p_1(v) + tr_1(v)}{p_0(v)^2}\right)$$

$$= -(\chi_1 + t\chi_2), \tag{S45}$$

and $q_2 = (1-t)p_2 + t\,r_2 + t(1-t)s$, we have that

$$J_2 = \Lambda^{-1}(\chi_1 + t\chi_2) + t\,\Lambda^{-1}D\Gamma + \Lambda^{-1}Dc \tag{S46}$$

so

$$D^\top \Lambda^{-1}(\chi_1 + t\chi_2) + t\,L\Gamma + Lc = r_2 - p_2 + (1-2t)s \tag{S47}$$

and since this holds for all t, this implies that

$$D^\top \Lambda^{-1}(\chi_1) + Lc = r_2 - p_2 + s \tag{S48}$$

$$D^\top \Lambda^{-1}(\chi_2) + L\Gamma = -2s,$$

thus we immediately know s and can find c through one linear solve. In total

$$I_2 = -\lambda_0^\top(r_3 - p_3) + \chi_3 + \int_0^1 \frac{1}{2}J_2^\top \Lambda J_2 - J_2^\top(\chi_1 + t\chi_2) + \Gamma^\top q_2 \mathrm{d}t, \tag{S49}$$

$$= -\lambda_0^\top(r_3 - p_3) + \chi_3 + \int_0^1 \frac{1}{2}(t\Gamma + c)^\top L(t\Gamma + c) - \frac{1}{2}(\chi_1 + t\chi_2)^\top \Lambda^{-1}(\chi_1 + t\chi_2)\mathrm{d}t + \Gamma^\top(p_2/2 + r_2/2 + s/6)$$

$$= -\lambda_0^\top(r_3 - p_3) + \chi_3 + \frac{1}{2}c^\top Lc + \frac{1}{2}c^\top L\Gamma + \frac{1}{6}\Gamma^\top L\Gamma - \frac{1}{2}\chi_1^\top \Lambda^{-1}\chi_1$$

$$- \frac{1}{2}\chi_1^\top \Lambda^{-1}\chi_2 - \frac{1}{6}\chi_2^\top \Lambda^{-1}\chi_2 + \Gamma^\top(p_2/2 + r_2/2 + s/6).$$

4. Combining orders

The above perturbation problem applies to general perturbations, but to compute the curvature we are only interested in a specific perturbation. In particular, calling $pr = \bar{W}(\rho_t, \rho_{t-\Delta t})$, $rq = \bar{W}(\rho_t, \rho_{t+\Delta t})$, and $pq = \bar{W}(\rho_{t+\Delta t}, \rho_{t-\Delta t})$, we have that the local Menger curvature is

$$\kappa(t) = \lim_{\Delta t \to 0} \frac{\sqrt{(pq + pr + rq)(-pq + pr + rq)(pq - pr + rq)(pq + pr - rq)}}{pq \cdot pr \cdot rq}, \tag{S50}$$

and moreover, we will show that in the limit

$$rq = \epsilon\sqrt{I_0^f} + \frac{\epsilon^2}{2}\frac{I_1^f}{\sqrt{I_0^f}} + \epsilon^3\left[\frac{1}{2}\frac{I_2^f}{\sqrt{I_0^f}} - \frac{1}{8}\frac{(I_1^f)^2}{(I_0^f)^{3/2}}\right] + \ldots, \tag{S51}$$

$$pr = \epsilon\sqrt{I_0^f} - \frac{\epsilon^2}{2}\frac{I_1^f}{\sqrt{I_0}} + \epsilon^2\left[\frac{1}{2}\frac{I_2^f}{\sqrt{I_0^f}} - \frac{1}{8}\frac{(I_1^f)^2}{(I_0^f)^{3/2}}\right] + \ldots, \tag{S52}$$

$$pq = 2\epsilon\sqrt{I_0^f} + \frac{\epsilon^3}{4}\frac{I_2^c}{\sqrt{I_0^f}} + \ldots, \tag{S53}$$

and hence

$$\kappa = 4(I_0^f)^{-3/4}\sqrt{\frac{I_2^f - I_2^c/4}{\sqrt{I_0^f}} - \frac{1}{4}\frac{(I_1^f)^2}{(I_0^f)^{3/2}}}, \tag{S54}$$

where we will now define the quantities I_0^f, I_1^f, I_2^f, I_2^b, and I_2^c.

To begin, consider the forward derivative,

$$\bar{W}(\rho_t, \rho_{t+\Delta t})^2 = \epsilon^2 I_0^f + \epsilon^3 I_1^f + \epsilon^4 I_2^f, \quad p = \rho_t + \Delta t\dot{\rho}_t + \frac{1}{2}\Delta t^2\ddot{\rho}_t, \quad q = \rho_t, \tag{S55}$$

as well as the reverse derivative

$$\bar{W}(\rho_t, \rho_{t-\Delta t})^2 = \epsilon^2 I_0^b + \epsilon^3 I_1^b + \epsilon^4 I_2^b, \quad p = \rho_t - \Delta t\dot{\rho}_t + \frac{1}{2}\Delta t^2\ddot{\rho}_t, \quad q = \rho_t. \tag{S56}$$

and the centered difference

$$\bar{W}(\rho_{t-\Delta t}, \rho_{t+\Delta t})^2 = \epsilon^2 I_0^c + \epsilon^3 I_1^c + \epsilon^4 I_2^c, \quad p = \rho_t + \Delta t\dot{\rho}_t + \frac{1}{2}\Delta t^2\ddot{\rho}_t, \quad q = \rho_t - \Delta t\dot{\rho}_t + \frac{1}{2}\Delta t^2\ddot{\rho}_t. \tag{S57}$$

We have that $r_1^f = r_1^r = 0$, and $p_1^f = -p_1^r$, as well as $\Lambda^f = \Lambda_r = \Lambda^c$, so that Λ and hence L does not change. Therefore

$$\begin{aligned} I_0^f &= \lambda_0^{f\top} p_1^f, \quad \text{where } L\lambda_0^f = p_1^f \\ I_0^b &= -\lambda_0^{b\top} p_1^f, \quad \text{where } L\lambda_0^b = -p_1^f \\ I_0^c &= 4\lambda_0^{c\top} p_1^f, \quad \text{where } L\lambda_0^c = 2p_1^f, \end{aligned} \tag{S58}$$

so that $I_0^f = I_0^b = I_0^c/4$, and $J_1^f = -J_1^b = J_1^c/2$.

Next, at first order

$$\begin{aligned} I_1^f &= p_2^\top \lambda_0^f - \sum_{e=(u,v)} \frac{J_1^f(e)^2}{4}\left(\frac{p_1(u)}{p_0(u)^2} + \frac{p_1(v)}{p_0(v)^2}\right) \\ I_1^b &= p_2^\top \lambda_0^b - \sum_{e=(u,v)} \frac{J_1^b(e)^2}{4}\left(\frac{-p_1(u)}{p_0(u)^2} + \frac{-p_1(v)}{p_0(v)^2}\right) \\ I_1^c &= 0, \end{aligned} \tag{S59}$$

showing that $I_1^f = -I_1^b$.

At second order, the contribution to I_2 from the term $-\lambda_0^\top(r_3 - p_3)$ satisfies $\lambda_0^{f\top} p_3 = \lambda_0^{b\top}(-p_3) = \frac{1}{4}\lambda_0^{c\top}(2p_3)$, so that in the combination $I_2^f + I_2^b - I_2^c/2$, they cancel.

Next, note that as $\chi_1^f = \chi_1^b$, $\chi_2^f = \chi_2^b$, $\chi_2^f = \chi_2^b$, and $\Gamma^f = \Gamma^b$, we therefore have $s^f = s^b$, and so $c^f = c^b$. Therefore $I_2^f = I_2^b$. There is no straightforward way to relate I_2^c to I_2^f however, so in total three linear systems must be solved to find the curvature. This is still substantially cheaper than solving a second order conic, or even a minimum cost flow problem.

B. Local relation between $\bar{W}$ and d_{TDD}

The diffusion distance between $p = p_0 + \epsilon p_1 + \epsilon^2 p_2 + \dots$, and $r = p_0 + \epsilon r_1 + \epsilon^2 r_2 + \dots$, is given by $d_{TDD}(p, q) = ||DL^\dagger(p - r)||$, with $q = tr + (1 - t)p$ being one of many possible paths between them that is a geodesic under the d_{TDD} distance. Under the metric $\bar{W}$, the geodesic path is given by

$$q = p_0 + \epsilon\left(tr_1 + (1 - t)p_1\right) + \epsilon^2\left(tr_2 + (1 - t)p_2 + t(1 - t)s\right) + O(\epsilon^3). \tag{S60}$$

For this to be a geodesic path under d_{TDD} as well, we would need that

$$||DL^\dagger(p - r)||_1 = \sum_{i=1}^{n} d_{TDD}(q_{t_i}, q_{t_{i-1}}), \tag{S61}$$

for all $0 = t_0 < \dots < t_n = 1$. However, we have that

$$d_{TDD}(q_{t_i}, q_{t_{i-1}}) = (t_i - t_{i-1})||DL^\dagger\left[(p - r) + \epsilon^2(1 - t_i - t_{i-1})s + O(\epsilon^3)\right]||_1. \tag{S62}$$

Taylor expanding for a single component, one has $|v + \epsilon^2 u + O(\epsilon^3)| = |v| + sign(v)\epsilon^2 u + O(\epsilon^3)$, and so we have that

$$\begin{aligned}\sum_{i=1}^{n} d_{TDD}(q_{t_i}, q_{t_{i-1}}) =& \sum_{i=1}^{n}(t_i - t_{i-1})d_{TDD}(p, q) + \epsilon^2 sign(DL^\dagger(p - q)) \cdot DL^\dagger s\left[t_i(1 - t_i) - t_{i-1}(1 - t_{i-1})\right], \\ =& d_{TDD}(p, r) + O(\epsilon^3).\end{aligned} \tag{S63}$$

Thus, at least to order ϵ^3, the unique geodesic under $\bar{W}$ is still a geodesic under d_{TDD}. Thus our curvature is truly a measure of how far the observed trajectory is away from the dissipation minimizing geodesic of d_{TDD}.

C. Fitting an empirical curve with kernel density estimation

Given a curve in the space of distributions on the flip graph, $p(t)$, we now have a way to compute the Menger curvature at a point, as a function of the first and second derivatives of the curve, $\kappa(t) = F(p(t), \dot{p}(t), \ddot{p}(t))$. However, we do not have access to the distribution, only empirical samples. We therefore wish to fit a smooth curve through these empirical samples, which we can then differentiate to compute the curvature. In the regime where samples are taken relatively far apart, but each sample is a good approximation to the true distribution, a spline can be fit through the samples [58]. However, in the regime where the distributions may be undersampled, we do not wish to fit a curve through each sample. Instead, given samples $p_1, \dots, p_N$, at times $t_1, \dots, t_N$, we take a kernel density estimation approach and say

$$p(t) = \sum_{i=1}^{N} a_i(t) p_i \tag{S64}$$

$$\text{with } a_i(t) = \frac{\exp\left[-\frac{(t-t_i)^2}{\sigma^2}\right]}{\sum_{j=1}^{N}\exp\left[-\frac{(t-t_j)^2}{\sigma^2}\right]},$$

so that $p(t)$ is an average of the empirical observations, weighted to primarily include samples within a range of σ away from t, and normalized to ensure $p(t)$ remains a probability distribution. Using automatic differentiation, it is then straightforward to find $\dot{p}$ and $\ddot{p}$ at any given t.

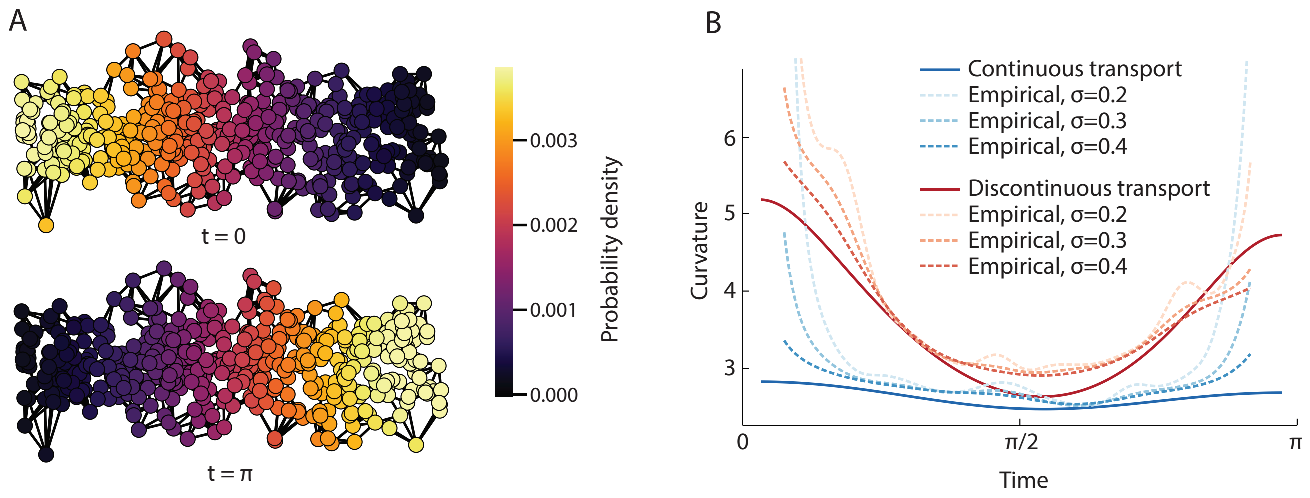

FIG. S9. Computing the Menger curvature from empirical samples. (A) Random network with initial and final probability distributions at times $t = 0$ and $t = \pi$. We will consider two different paths, p_c and p_d between these distributions. (B) Menger curvature of the continuous path, p_c and the discontinuous path p_d, along with the empirical approximations from finite sampling.

D. Numerical validation

In order to validate the curvature framework, we introduce here a model system. We create a network by sampling 500 points uniformly in $x \in [-0.5, 0.5]$ and as $\mathcal{N}(0, 0.1)$ in y, and connecting each point to their 7 nearest neighbors. We consider two different paths on this graph,

$$\begin{aligned} N(x,y) &= e^{-3(x^2+y^2)} \\ p_c(t,x,y) &\propto N(x-\cos t, y) \\ p_d(t,x,y) &\propto (1+\cos t)N(x-1,y) + (1-\cos t)N(x+1,y), \end{aligned} \tag{S65}$$

where p_c and p_d are only defined for x and y where there is a vertex, and there is a proportionality factor ensuring that p_c and p_d always remain normalized. Intuitively, p_c represents a continuous path, where probability density is transported from the left hand side of the network (Fig. S9A) at $t = 0$ to the right hand side at $t = \pi$ by continuously shifting it from left to right. In contrast, p_d represents a discontinuous path where probability density instantaneously switches from the left side to the right side. Both distribution agree at initial and final time points, $p_c(0) = p_d(0)$, $p_c(\pi) = p_d(\pi)$.

Intuitively we would expect p_c to have lower curvature than p_d, and we confirm this by computing the curvature exactly from the functions p_c and p_d, Fig. S9B. Moreover, we would expect the curvature of p_c to be relatively constant in time, whereas we would expect the discontinuous path to have largest curvature at the start and end times, where probability density must appear in an area with previously very low probability, Fig. S9B. Note that the reflection symmetry around $t = \pi/2$ is broken by the normalization and the random distribution of points.

Now we draw finite samples from this distribution. At time intervals of $t = 0.1$, we draw 8000 samples from each distribution, a notable undersampling. From this, we use kernel density estimation to construct a continuous curve and compute the Menger curvature. We approximately recover the curvature, Fig. S9B, although taking too small of a σ value leads to spurious oscillations, especially around the end points.

E. Zebrafish curvature

After computing the pairwise distance matrix between 90 time points of the zebrafish embryo, we recover a 1D manifold that is parameterized by time, Fig 2D. We find that development changes the topological structure, and that these developmental changes are more notable than finite sampling effects as minimal fluctuations around this path are present in the embedding. The path that this manifold takes is not a straight line in the embedding. Contrast that with the developmental trajectory of a 2D fly wing, which was found to take an optimal path from the initial distribution to the final one [31]. To analyze where the path is least optimal, without the distorting effects of a low

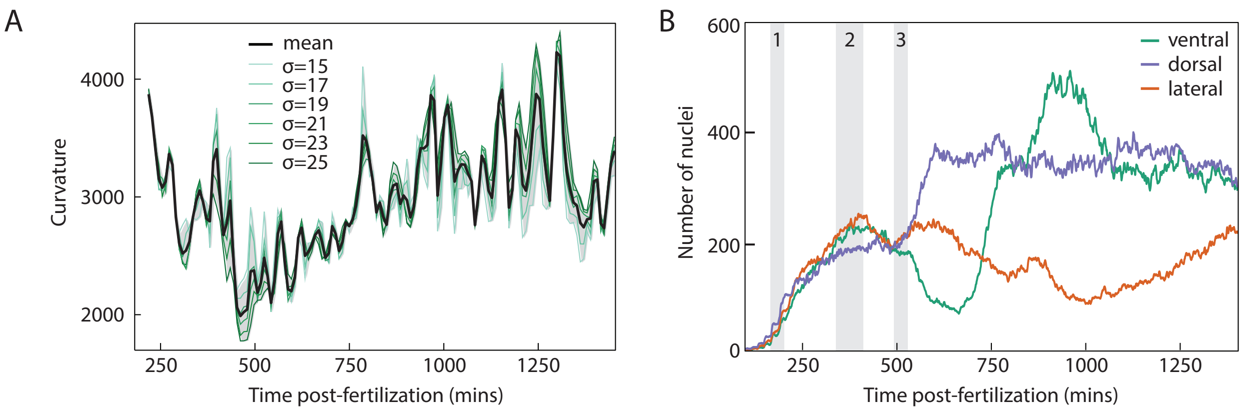

FIG. S10. Topological efficiency of developmental trajectory is revealed by a curvature computation for zebrafish embryogenesis. (A) To analyze the topological trajectory without using a low dimensional embedding, which will necessarily distort the data, we compute the curvature, a measure of how efficient the topological path is (higher curvature is less efficient). To compute the curvature, kernel density estimation was used with different smoothing parameters $\sigma = 15, \ldots, 25$, from which a mean value was computed (black). (B) Number of cell nuclei in dorsal ventral and lateral regions of the embryo, adapted from Ref. [19] with permission. Three symmetry breaking events (gray regions) were identified by Ref. [19], namely: (1) symmetry breaking in cell divisions, (2) symmetry breaking in cell density, (3) symmetry breaking in morphology. Following cell numbers in each region after these initial symmetry breaking events provides a partial way to identify developmental changes, albeit limited as it only tracks cell numbers and not cell rearrangement or topology.

dimensional embedding, we compute the curvature. After starting at a large curvature, the curvature drops at around 500 m.p.f., corresponding to the straightest part of the MDS embedding, before increasing again, Fig. S10A. Spikes in curvature can correspond to abrupt changes the topological trajectory, and could correspond to abrupt developmental changes. To further investigate this, we can compare this to the nuclei counts in three domains, dorsal, ventral, and lateral, that were used in Ref. [19] to identify symmetry breaking events, Fig. S10B. The first event identified by Ref. [19], is symmetry breaking in cell divisions, which happens early on when the curvature is high. The next corresponds to symmetry breaking in cell density which happens at a local maximum of curvature. After this, the curvature drops to it's lowest point as the total number of cells remains flat, with mostly rearranging rather than cell division occurring, before the final identified symmetry breaking event of the first morphological differences between regions. The next notable spike in curvature comes at around 800 m.p.f., where a rapid growth of cells in the ventral region stops, marking the end of some particular developmental phase and the beginning of another. Subsequent spikes in curvature become hard to associate with a particular developmental change, as identified by coarse grained nuclei counts, as by this point, many independent processes are occurring across the embryo.

V. BOOTSTRAPPING DISTANCE CALCULATIONS

Whilst the MDS embedding shows that different species of biofilm have different topological distributions, we would like to test statements of that form statistically. Consider the example of comparing *E. coli* and *V. cholerae*, where we have 15 *E. coli* experiments and 15 *V. cholerae* experiments. To compare the two species, we can combine all 15 *E. coli* experiments into a single observed distribution and compute the distance with the combined 15 *V. cholerae* experiments. How can we know whether the resulting distance is statistically significant? In particular, due to finite sampling effects, we would expect there to be a non-zero distance between even 15 combined *E. coli* experiments and another independent set of 15 combined *E. coli* experiments; the empirical distributions would not perfectly match even though there is no underlying structural difference. However, by comparing against a null distance, we are able to perform significance tests on the hypothesis that the distance between two species is non-zero.

Assume for a moment the null hypothesis, that there is no structural differences between *E. coli* and *V. cholerae*. In this case, there is nothing special about splitting the 30 experiments into 15 *V. cholerae* and 15 *E. coli* experiments, as they are all structurally identical. There are many other ways to divide these 30 experiments into two, for instance one group with 10 *E. coli* and 5 *V. cholerae* experiments, and another group with 5 *E. coli* and 10 *V. cholerae* experiments. In fact, there are $\binom{30}{15}/2 = 77558760$ ways of dividing the 30 experiments into two equally sized groups. If the null hypothesis holds, there is nothing special about the distance between 15 *V. cholerae* and 15 *E. coli* experiments, as compared to distances resulting from all the other ways to split the experiments. However, when we compute the

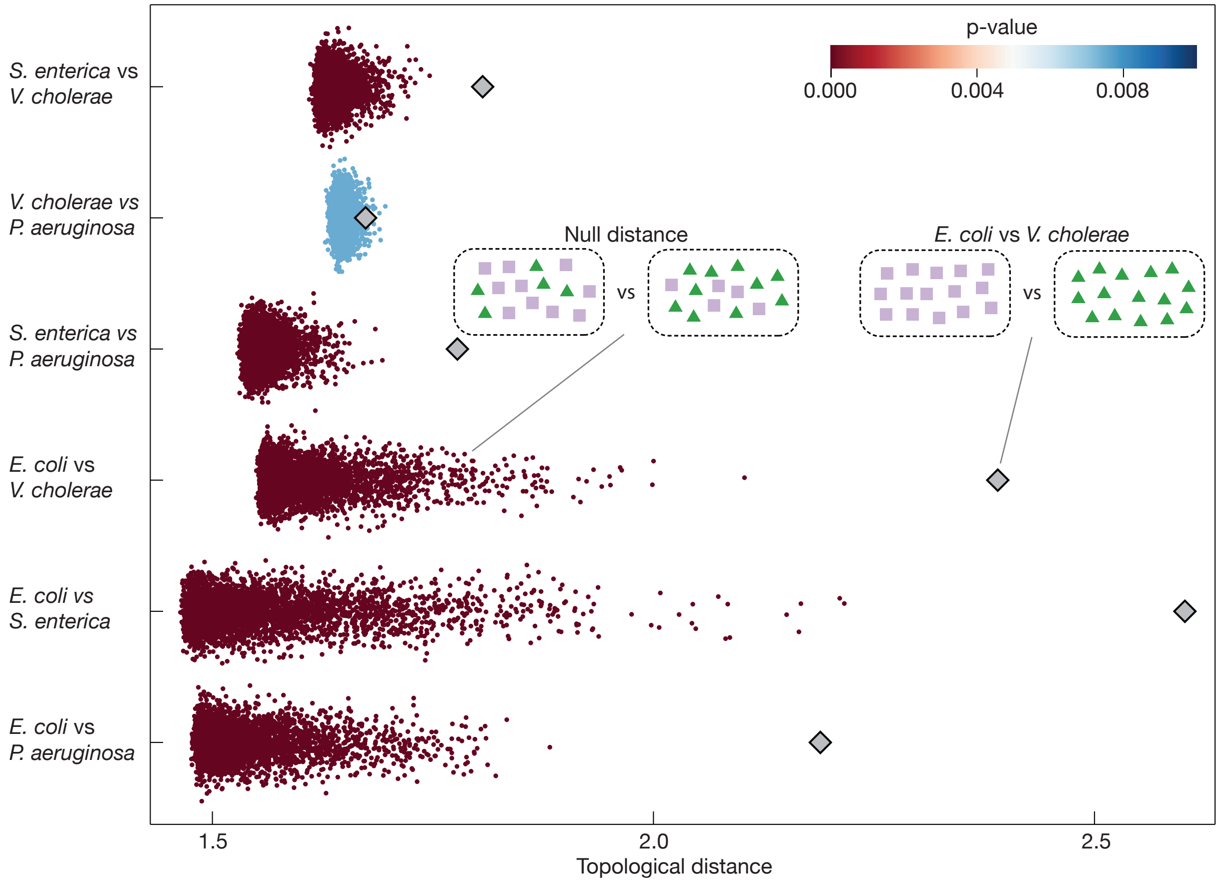

FIG. S11. Topological distance between different bacterial species is statistically significant at $p < 0.01$. For each pair of species the topological distance was computed between the combined distribution of 15 experiments from the first species and the combined distribution of 15 experiments from the second species (gray diamonds). This was compared to a null distance which was formed by randomly splitting the 30 combined experiments into groups of two, which was done 2000 times (points, colored by p-value). In all cases the distance between species is greater than 99% of null distances.

distances between 2000 randomly sampled different ways of splitting the 30 experiments, the *V. cholerae* vs *E. coli* split is larger than any of them, Fig. S11.

We can repeat this for all match ups of different biofilm species, finding for all of them that every pairwise combination of species is statistically significantly different for at p-value $p < 0.01$, meaning that the pairwise distance when split by species is bigger than 99% of random splittings. We therefore can conclude that every one of the 4 species considered has a topologically distinct structure from any of the others. Whilst shown to be topologically distinct, *P. aeruginosa* and *V. cholerae* form biofilms that are more similar structurally than any other pair of species, with both having similar aspect ratio cells.

We can also apply this analysis to the different regions of the juvenile zebrafish brain, as was done in Fig. 2 of the main text. Nuclei within the brains were categorized through a semi-automated histological approach into 9 major brain regions [13], and as a point of comparison, we also divided the nuclei into 9 regions along the principal head–tail axis, with each region containing an approximately equal number of points. With only 5 experiments available, we can not say that all major brain regions are statistically different, but at $p < 0.05$ we can say that 32 (out of 36) pairwise comparisons are significantly different. In comparison, for the regions created by partitioning along the major axis, only 8 pairwise comparisions are statistically significant. Further, all of these 8 comparisons involve region 1, which is effectively the olfactory epithelium and telencephalon regions combined, whereas all other partitioned regions are a mix of several different major brain regions.

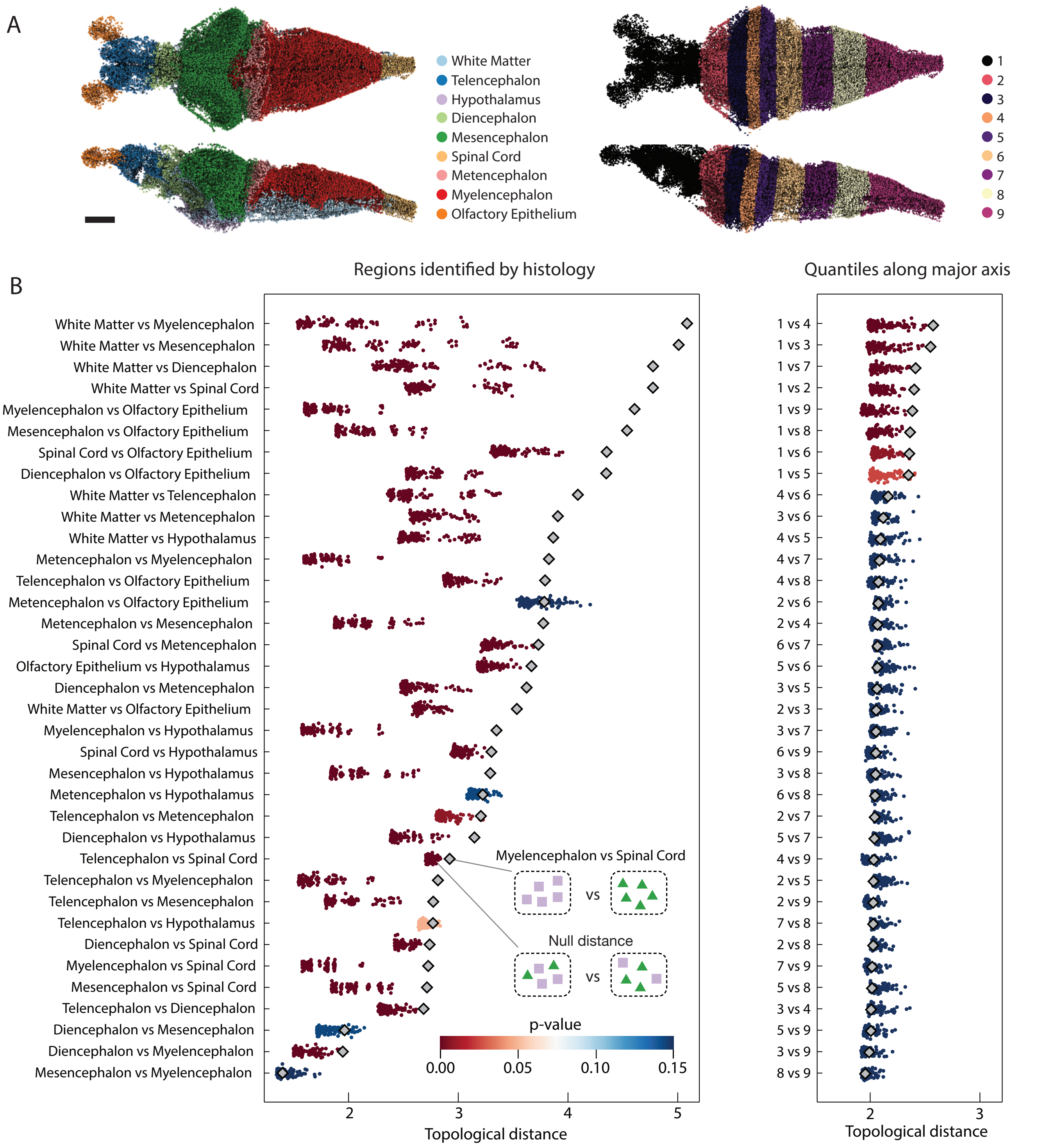

FIG. S12. Regions of the zebrafish brain identified by histological imaging differ in a statistically significant way, in contrast to regions created by partitioning along the major axis. (A) Zebrafish brain nuclei from Ref. [13] colored by the 9 major brain regions (left) and by partitioning into 9 regions with equal nuclei along the major axis. Scale bar is 100μm. (B) For every pair of zebrafish brain regions, the topological distance was computed between the combined distribution of the first region taken from 5 experiments and the combined distribution of the second region taken over 5 experiments (gray diamonds). This was compared to a null distance which was formed by randomly splitting the 10 regions (2 from each experiment) into two groups of 5, which was done all 126 possible times (points, colored by p-value). This was done for the 9 major brain regions identified by Ref. [13] with histological imaging analysis (left), and for 9 regions created by partitioning along the major axis (right). With only 5 experiments, not all pairs of regions can be shown to differ in a statistically significant way. However, for the major brain regions, 32 pairs (out of 36) differ at $p < 0.01$, whereas only 8 pairs do for the major axis partitioning.

VI. DETAILS OF DATASETS USED

Biofilms. Bacterial biofilms are multicellular communities of cells that grow on surfaces and are held together by an extracellular matrix [59], and are among the most abundant forms of microbial life on earth [60]. We make use of the segmented images of bacterial biofilms from Ref. [32], with 15 biofilms imaged from the four species, *E. coli, V. cholerae, S. enterica, P. aeruginosa.* The segmented colony contains the size, position, and orientation of every cell within the colony [32], here we use only the cell centroid position. The biofilms are imaged whilst growing, and for each experiments we have a number of time points. We use only the images when the biofilm contains between 1500 and 3500 cells, typically resulting in around 4 images per biofilm. We combine all the motifs from each image of the same biofilm into one distribution. After calculating the distance $d_{\mathrm{TDD}}(A, B)$, for all pairs of biofilms A, B, we embed this distance matrix in 2D using multi-dimensional scaling, and can color according to single cell properties, Fig. S13. We see that cell aspect ratio, and related quantities like cell length, color the principal component of the manifold on which the data lie.

Zebrafish brain. Juvenile zebrafish brains were imaged by micro-CT tomography and the spatial position of all nuclei were segmented in Ref. [13]. In total there are 5 experiments available, each containing around 80,000 nuclei. In addition to nuclei segmentation, Ref. [13], applied a semi-automated histological approach to assign each nuclei to one of 9 major brain regions. Motifs were computed prior to assigning the into major brain regions, so if the central vertex of a motif lies in one region, that motif is assigned to that region even if it contains vertices in different regions.

For the 5 experiments available, and for every major brain region, we take a combined motif distribution using the nuclei assigned to that region across all experiments. Using these distributions, we compute the pairwise distance matrix between every brain region, Fig. S14A. We see that, for example, the distance between white matter and Myelencephalon is over 10 times the difference between Mesencephalon and Myelencephalon. Indeed, from Fig. S12, we see that the distance between white matter and Myelencephalon is statistically significant at $p < 0.01$, whereas the distance between the Mesencephalon and Myelencephalon is not statistically significant at this (limited) level of data.

In contrast, taking the 9 regions along the major axis, across experiments, the embedding does not show systematic variation across regions, with variation between experiments playing as important of a role, Fig. S14C.

Embryo development. A developing zebrafish was imaged using lightsheet microscopy and the position of all nuclei were determined from around 100 to 1500 minutes post fertilization by Ref. [19]. In total, 900 time points were imaged at regular intervals separated by 90s. For the MDS embedding, the 900 time points were split into 90 regions, each containing 10 subsequent time points, and the pairwise distance was calculated between these regions, as shown in Fig. S15. For the combined embedding, only 6 of these 90 regions were used, to illustrate different developmental stages.

The same group imaged a developing *D. melanogaster* embryo with lightsheet microscopy and again collected the position of all nuclei from 120 to 690 minutes post fertilization by Ref. [12]. In total, 191 time points were imaged at regular intervals separated by 180s. For the combined embedding, only 7 time points were used. All times for both the zebrafish and *D. melanogaster* are measured in minutes post-fertilization (m.p.f.).

We also made use of two additional experiments of embryonic development, the ascidian *P. mammillata* and the worm *C. elegans.* Detailed images of the developing ascidian were collected by Ref. [40] across 6 experiments. We used all images containing over 250 cells, and took the cell barycenters as input points for the Delaunay. For *C. elegans*, 46 experiments were performed and nuclei positions obtained by Ref. [39]. For each of these experiments, we an image which occurred at around the 350 cell stage.

Human Cancer Organoid. The mechanical properties and physical arrangement of human cancer cells determine their ability to invade surrounding tissues [41]. In total, 18 human cancer organoids were imaged by Ref. [41] at the 21 day stage, comprised of around 100-400 cells, with the nuclei detected. We used the spatial location of the nuclei for the combined embedding.

Random packings. Random packings of spheres and ellipsoids were generated using the event driven packing code from Ref. [22]. This created a jammed periodic packing of 10,000 particles, where the centroids of the particles were used for subsequent analysis. The periodicity allows us to compute the motif for every point without boundary effects, since every point can be considered to be in the bulk. Simulations were performed for spheres, $1:1:4$, $1:4:4$, and $1:2:3$ aspect ratio ellipsoids. Simulations were also performed for a polydispere mixture of spheres with radii in ratio $2:3$, and with equal numbers of each.

Diffusion limited aggregation. In order to model the process by which particles combine in order to form an aggregation, for instance in dust or soot, a mathematical model known as diffusion limited aggregation was introduced and has been widely studied [61]. Simulations of diffusion limited aggregation were performed with code from Ref. [62] with the default parameters.

Glassy material. Under certain conditions, liquids can be cooled down to a solid-like state without forming local crystaline order. Understanding the nature of this transition, as well as the properties of such glassy systems is a central

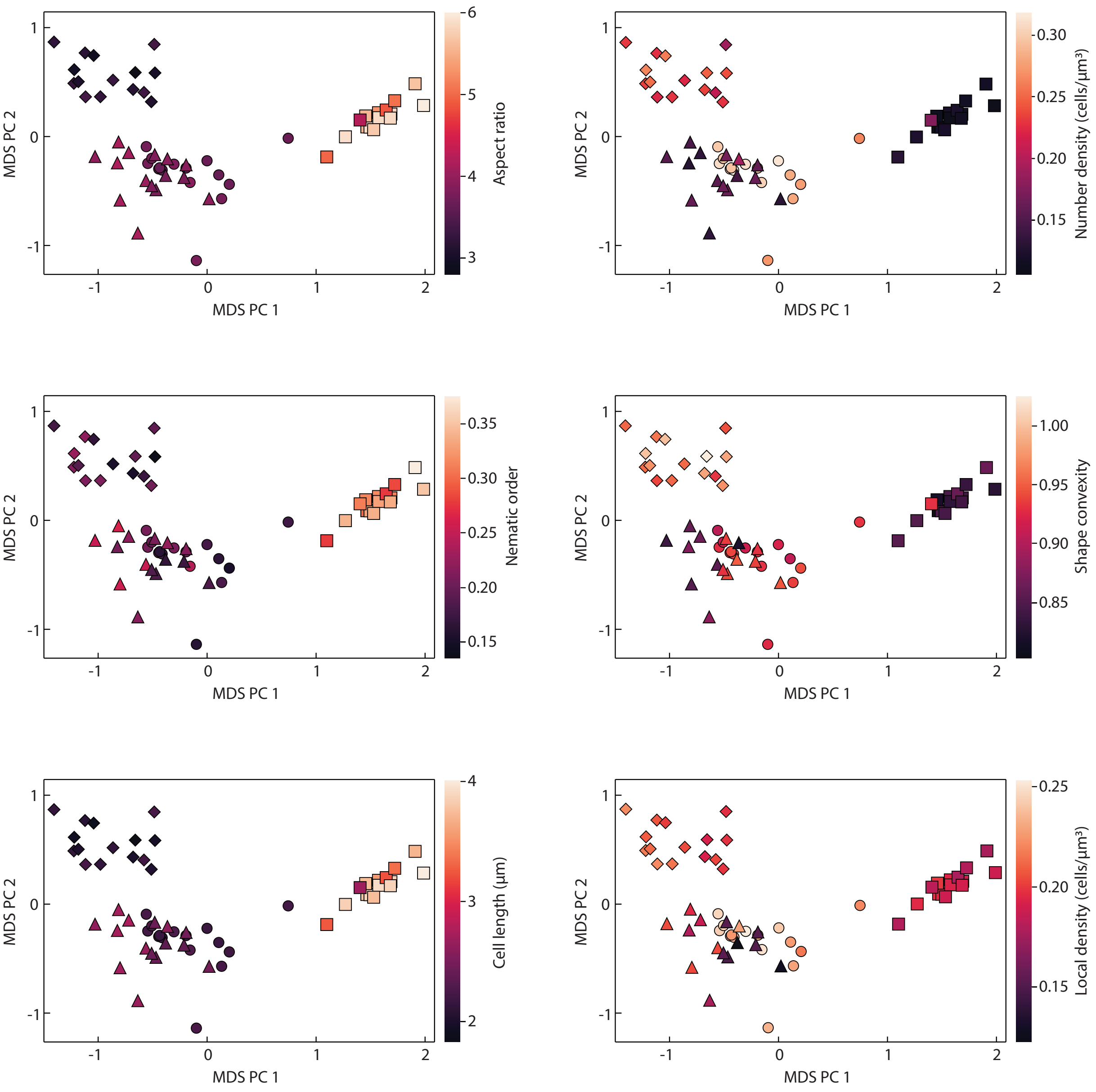

FIG. S13. MDS embedding of biofilm data colored by single cell properties finds that aspect ratio explains the data manifold. For every biofilm experiment, the cell aspect ratio, cell length, and shape convexity were calculated for each cell, and the mean was taken to create an average value for the experiment. The remaining parameters were computed by drawing a ball for every cell of radius 2 μm and computing the nematic order parameter, the local number density (cells per volume), and the density (volume occupied by cells), within that ball. These were again averaged to create a value for each experiment. Coloring by aspect ratio and the related quantities of cell length and nematic order parameter, provides a consistent coloring along the data manifold (left). Coloring by other quantities does not (right). This suggests that differing cell aspect ratio is the principal reason for topological differences between colonies.

challenge in physics [23]. Simulations of a glassy material specifically a 80:20 Kob–Andersen-type Lennard–Jones mixture were performed by Ref. [23] with 4096 particles in a periodic box. We used the final time point of simulations performed at temperature $T = 0.44$, well into the glassy phase.

Star positions. The positions of the nearest 110,000 stars to earth were taken from the HYG star database [63], which collates three previous databases, Refs [30,64-65]. Within this database, only the stars with known position, and not just angle, were used. In the computation of the motif distribution, motifs at the edge of this dataset were removed, as this is not a physical boundary.

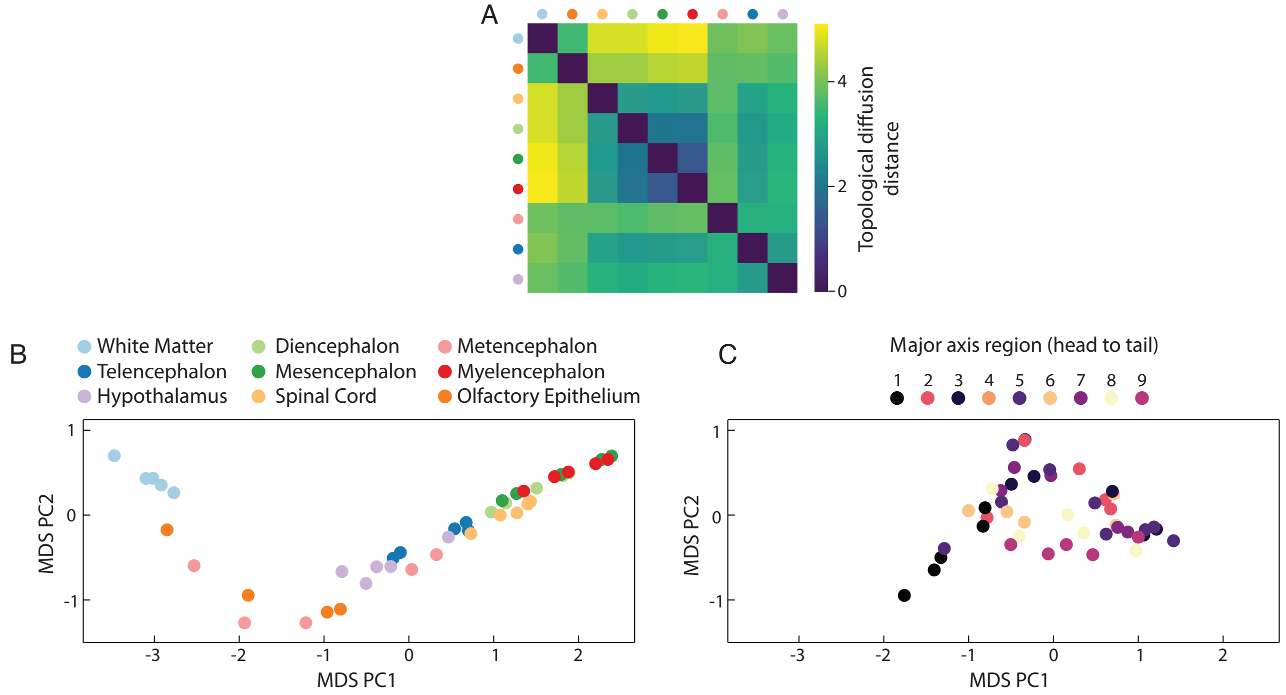

FIG. S14. Histologically identfied regions of the juvenile zebrafish brain show systematic topological differences. (A) Combining all experiments to compute a distance between regions, we see that some regions are far more topologically different than others. For instance, the white matter region is very different than the Myelencephalon region ($p < 0.01$), but the Myelencephalon region is topologically similar to Mesencephalon region (no statistically significant difference). (B) Embedding all regions of the brain for each experiment separately reveals consistent differences between brain regions across experiments, as well as a broadly one-dimensional manifold along which regions vary. (C) In contrast, dividing into 9 equally sized regions based on the distance along the major axis reveals no significant difference between regions.

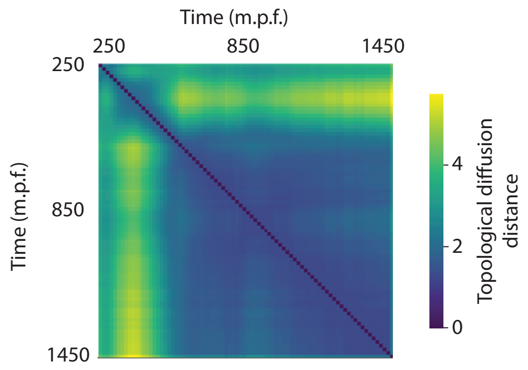

FIG. S15. Topological distance matrix for the zebrafish embryo showing pairwise distances between 90 time points. The resulting MDS embedding of this distance matrix is shown in Fig 2D.

A. Combined embedding and residual variance

When embedding a (non-Euclidean) distance matrix into a lower dimensional Euclidean space, we must ensure that the lower dimensional representation is not substantially distorting the true distance matrix. To do so, we can compare the true topological distance matrix with the distance matrix of the embedding, calculated by measuring the pairwise Euclidean distances of the embedding. We see that the Euclidean distances of the embedding remain faithful to the true distances, Fig. S16. The primary distortion is that some similar systems are forced closer together in the embedding than they actually are in topological distance, which can be improved by taking a higher dimensional embedding, Fig. S16. This distortion can occur when finite sampling effects mean that identical distributions are a

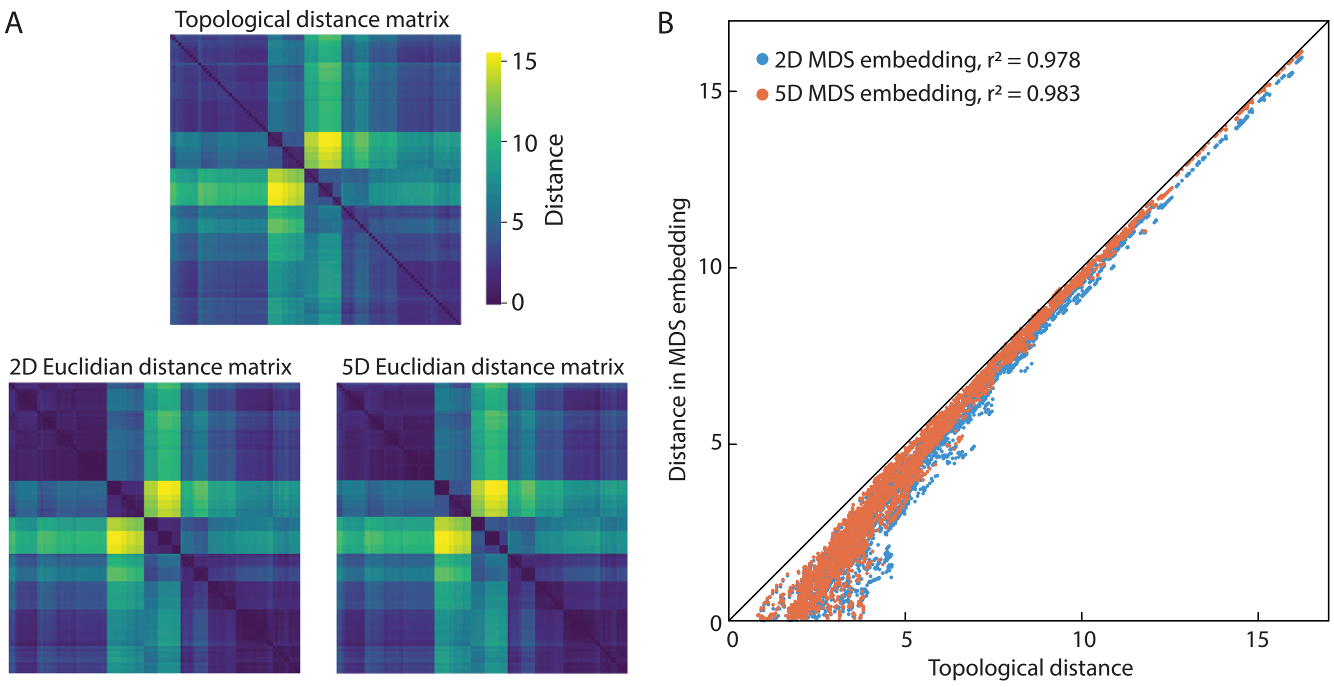

FIG. S16. Topological distance matrix is not substantially distorted by embedding into Euclidean space. (A) The topological distance matrix for living and non-living systems (top) was embedded into Euclidean space with MDS. Computing the Euclidean distance matrix of the resulting MDS embedding, largely preserves the distance structure (bottom left for 2D, bottom right for 5D). (B) Topological distance plotted against the Euclidean distance for the MDS embedding shows that the embedding preserves the distance structure. The main discrepancy occurs for similar regions which are forced closer together in the low dimensional embedding than they are in topological distance. We see that embedding into a higher dimensional Euclidean space (2D to 5D) improves the fidelity of the distance matrix, as measured by the correlation coefficient between topological distances and Euclidean distances of the MDS embedding ($r^2 = 0.978$ for 2D embedding, $r^2 = 0.983$ for 5D embedding).

non-zero topological distance away from each other, but the MDS embedding places them close together, since they are similarly distant to all other samples. Even for a 2D embedding, the correlation coefficient between topological distances and Euclidean distances in the embedded space is 0.978, demonstrating that the embedding quantitatively preserves the distance structure.

B. Comparing living and non-living systems

From the combined embedding in Fig. 3, we see that the region formed by taking the convex hull of all living systems only contains one non-living system in a 2D embedding, and contains no non-living systems in a 3D embedding.

Additionally, we can test whether there exists a linear hyperplane separating living and non-living systems by training a support vector machine on the embedding data. We find that in a 7 dimensional MDS embedding, a linear hyperplane exists which correctly classifies all points except the star database. Moreover, using a support vector machine with a non-linear kernel (exponential radial basis function), we can train a function that correctly classifies all but the star database and organoid data from a 2D MDS embedding, or all but the star database and two irregular ellipsoid packings from MDS components 2 and 3, Fig. S17.

C. Motif distribution analysis

In addition to the combined embedding and distance computation, it is of interest to investigate how the motif distributions vary. It is hard to visualize a distribution over the total space of motifs, instead we can compute simpler statistics of the distributions and study these. For instance, we can compute the mean motif size, which counts on average the number of tetrahedrons in a motif, as well as computing the variance of this quantity. We find that these systematically vary across different systems, Fig. S18. Whilst some of the features of the combined embedding are

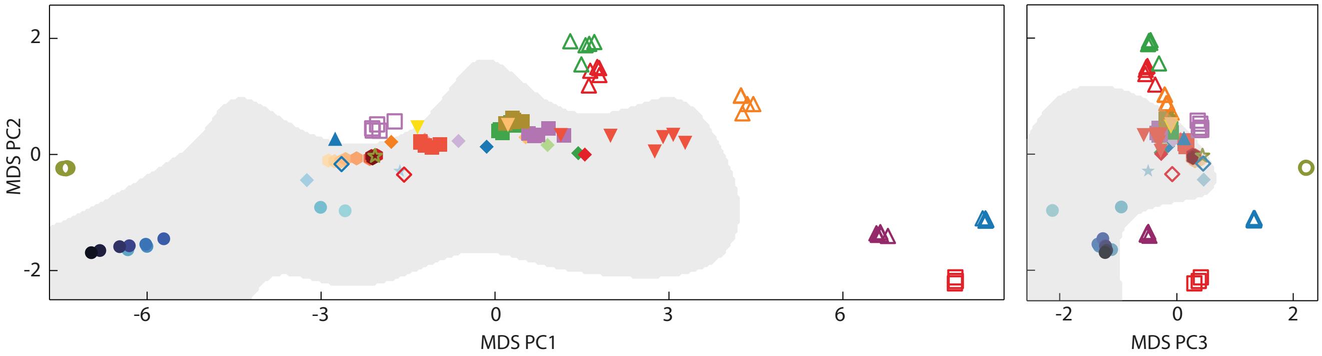

FIG. S17. Support vector machine with a non-linear kernel trained on the combined MDS embedding can classify living and non-living materials. From the first two components of the MDS embedding (left) a support vector machine with a single exponential radial basis function non-linear kernel was trained to classify living (closed symbols) and non-living (open symbols) systems. The region it assigns to living systems is shown in gray, and gets the classification correct with the exceptions of the galaxy, polyurethane foam, and fluid foam data sets. Repeating this procedure using components 2 and 3 of the MDS embedding (right) the support vector machine is similarly able to correctly classify points with the exception of the galaxy, polyurethane foam, and fluid foam data sets and one of the irregular ellipsoid packings (for full symbol legend see Fig. 3).

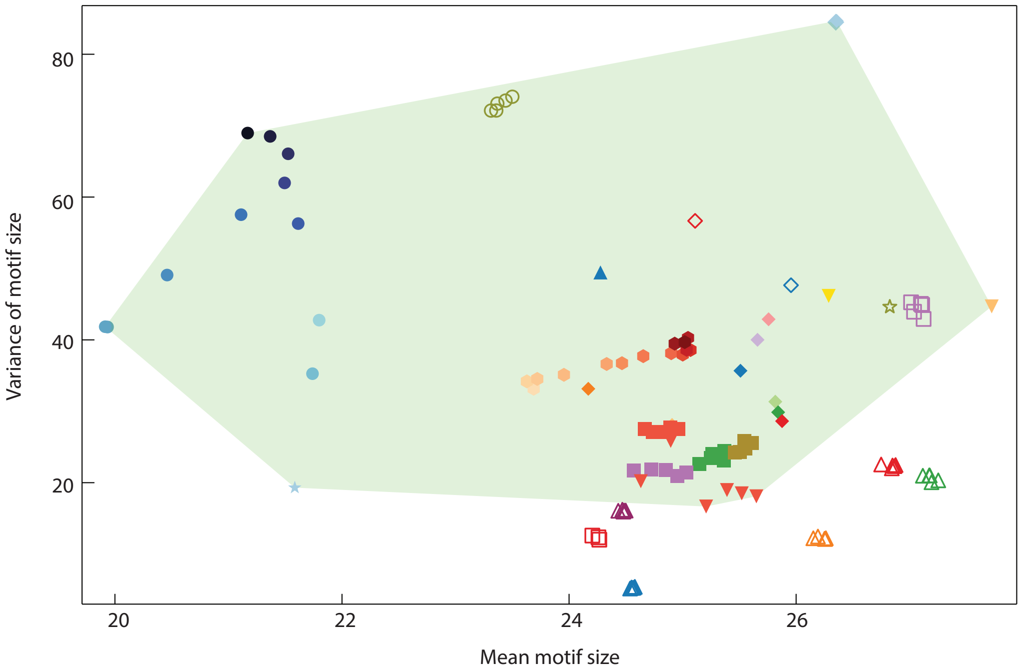

FIG. S18. Plotting mean motif size against variance of motif size reveals systematic differences across systems. Symbols are the same as main text Fig. 3, convex hull of living systems is shown in green. Multiple realizations of the same process, such as Poisson-Voronoi (purple squares) show that these statistics are not substantially affected by finite sampling effects.

also present in this moment embedding, the convex hull of living systems contains a number of non-living systems, Fig. S18. Moreover, systems that are only somewhat different in the mean and variance of motif sizes are in actuality very different according to the TDD distance, for instance the polydisperse packing and the biofilms, Fig. S18. Naturally grown vs randomized cell packings We hypothesize that a contributing factor as to why living systems are

topologically distinct from non-living systems is due to the manner in which they are grown. For instance, bacterial biofilms have a core of nematically aligned cells which forms through a growth induced buckling instability followed by the further elongation and dividing of cells [35, 66]. A random granular packing of similarly shaped ellipsoidal particles would not have this structure. However, another important contribution to the topological distribution comes from the cell geometry, including cell shape, size, and polydispersity, and this contribution is independent of whether the material was formed through growth or through a non-active process such as a random packing.

In order to demonstrate that the topological properties of a living system systematically depend on the formation process, we will take a living system and compare the topological structure to a system with the same cell geometry, but with randomized cell positions, representing a random packing. We will use the zebrafish brain dataset [13] as an example system to test on.

Particle representation. As with many the systems that we have considered here, we do not have the exact cell shape, only the nuclei position, and the corresponding Voronoi cell. For a more realistic cell shape, we will approximate this Voronoi cell by a multisphere particle [67]. Each cell is then represented as a collection of soft spheres $S_1, \ldots, S_k$ which interact with each other through springs of rest length l_{pq} for spheres p and q. To initialize the shape, we start with k spheres of radius r, where k is the number of neighbors of that Voronoi cell, and r is 1/6 the median distance of the centroid to its neighbors in the Delaunay. We then take the Voronoi cell as quadratically confining the spheres, so if the centroid of sphere S_i is distance ϵ from some face of the Voronoi cell, and $\epsilon < r$ where r is the radius of S_i, then there is an energetic penalty of $\frac{1}{2}(\epsilon - r)^2$. Such penalties, when summed over every confining face result in an overall confining potential B_i for sphere S_i. In order for the spheres to fill out the Voronoi cell, we consider an interaction term $I_i = \min_{j\neq i} r_{ij}$, with r_{ij} being the distance between centroids of S_i and S_j. Thus, I_i is simply the minimum distance of S_i to any another sphere, hence maximizing I_i discourages spheres from being too close. In total we then minimize a functional $E_{init} = \mu \sum_i B_i - \sum_i I_i$, where μ is a parameter tuning the relative effects of confinement vs mutual repulsion which we set to be $\mu = 100$. After minimizing the functional, if the median nearest neighbor distance is greater than $2r$, meaning the spheres are not touching, we increase the radius of all spheres by 10% and minimize again, if the median neighbor distance is less than $1.5r$, meaning notable overlap, we decrease the radius of all spheres by 10%, and minimize again, repeating this until a consistent initialization has been reached. We then make a note of the new pairwise distance between each sphere, say l_{ij}, and connect each pair of spheres with a spring (of stiffness 1) of that length. Upon any deformation of the particle, the internal spring energy is now $I = \sum_{i<j} \frac{1}{2}(r_{ij} - l_{ij})^2$, Fig. S19C

Bounding region. To confine the particles, we introduce a potential representing a bounding box. If a sphere S_i has it's centroid within a distance ϵ of a bounding wall, where $\epsilon < r_i$, then there is a potential of $\phi_i = (r_i - \epsilon)^2$, leading to a total confining potential of $T = \sum_i \phi_i$ where the sum is taken over all spheres from all particles, Fig. S19A.

Interaction energy. Consider now two particles, p and q consisting of spheres $S_1^p, \ldots, S_{k_p}^p$ of radius r_p and $S_1^q, \ldots, S_{k_q}^q$ of radius r_q respectively. We define the interaction energy between them as

$$V_{pq} = \sum_{1\leq i\leq k_p,\ 1\leq j\leq k_q} U_{ij}, \tag{S66}$$

where

$$U_{ij} = \begin{cases} (r_i + r_j - r_{ij})^2 & \text{for } r_i + r_j \geq r_{ij} \\ 0 & \text{else} \end{cases} \tag{S67}$$

with r_{ij} the distance between the centers of S_i^p and S_j^q. This poentital enforces a soft repulsion between particles, Fig. S19B.

Overall potential. The overall potential energy combines the internal multisphere particle spring energy, the interaction between particles, and the bounding box potential, so

$$E = \sum_p I_p + \sum_{p<q} V_{pq} + T. \tag{S68}$$

Starting from some initial condition, we will use gradient descent to find the nearest local minima of this function to find the final particle positions.

Initial relaxation. By construction, the initial particle positions before the randomization of cell positions, has zero internal energy, but there might be some slight overlap of spheres from different particles. We therefore relax the cell positions without reshuffling, observing slight movement which does not result in a substantial topological difference, Fig. S21A.

Randomization. To destroy the local structure generated through growth, whilst preserving particle geometry, we randomly permute particles. Specifically, if a particle which corresponds a nucleus at position $\boldsymbol{x}$, is sent to a new

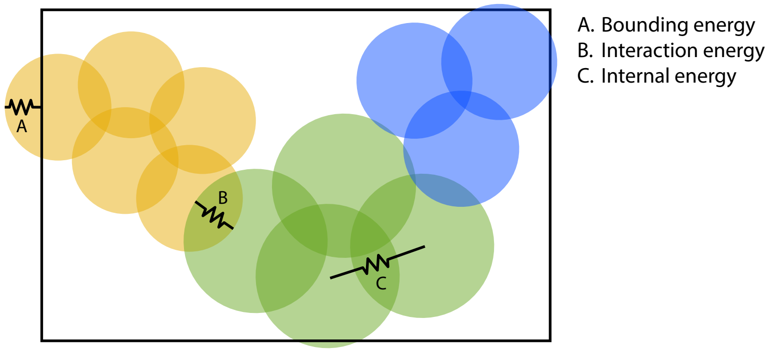

FIG. S19. Diagram showing select relevant interaction energies which contribute to the overall potential of the multisphere particle packing. (A) When a sphere belonging to the yellow particle extends past the bounding box, it incurs a spring like potential energy representing a confinement effect. (B) When a sphere from the yellow particle overlaps with a sphere from the green particle, it incurs a spring like potential energy representing a soft volume exclusion effect. (C) When two spheres from the green particle are deformed from their relative initial positions, it incurs a spring like potential energy representing a soft particle deformation penalty.

point $\boldsymbol{y}$ which corresponds to a different nucleus, then every sphere which makes up the particle is translated by $\boldsymbol{y}-\boldsymbol{x}$. A random rotation of the particle around $\boldsymbol{y}$ is then performed to remove any orientational order. From this permuted initial condition, we use gradient descent to find the nearest local minima the overall potential, which approximates a random packing. We do not permute points that are on the boundary, as their geometry may be unphysical. Instead we leave them in place, but allow them to deform during the relaxation to a local minima after the permutation occurs.

Identification of new nuclei. Each particle corresponds to a nuclei, so after the particle has been deformed, we need a new nuclei position in order to perform the topological analysis. To do so, we connect the nuclei to every sphere centroid with springs that are at their rest length for the initial particle configuration. After the particle has been deformed, the new nuclei position is calculated by minimizing the spring energy, allowing the nuclei to move with the particle.

Overall protocol. Due to the computational complexity of minimization growing quadratically with the number of points, and the large number of points for the zebrafish data, we do not permute the full dataset. Instead we draw a box of length 40μm around a randomly selected interior point and perform the permutation on this box, noting the initial point locations, the point locations after relaxation without permutation, and the point locations after permuting then minimizing the potential. An visualization of the procedure is shown in Fig. S20. We repeat this 12 times for each zebrafish experiment.

Results. After performing the permutation, we combine motifs across the same zebrafish experiment, and compute the pairwise distance between experiments both with and without the permutation. We see in Fig. S21B that there is a systematic difference between the relaxed points and the permuted points, indeed they are separated by the first principle component. This is despite the fact that for every point in the embedding corresponding to a permuted point, there is another point with identical particle geometry corresponding to the relaxed system. We also can combine all motifs corresponding to the permuted points and add the to the combined phase diagram. The combined system moves away from the bulk of the living systems, but remains within the convex hull of living systems, Fig. S21C. Interestingly the closest point to the permuted system is the industrial foam, the sole non-living system that crosses into living region. A conjecture would be that this reflects how the foam has a similar polydispersity, particle shape, and soft interparticle interactions as living systems, but lacks the growth mechanism, and hence removing growth makes a living system more similar to the foam. This remains an intriguing possibility for future research.

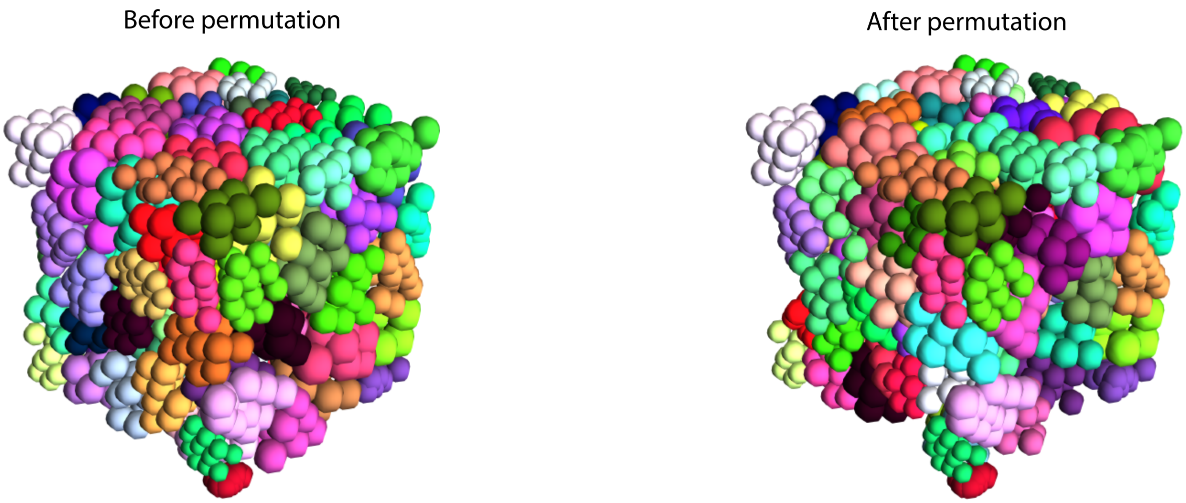

FIG. S20. Multisphere representation of a section of juvenile zebrafish brain data before and after permutation of the points. Cells are approximated by multisphere particles, different spheres belonging to the same particle are colored the same. The particle distribution before permutation, but after relaxation (left) and after permutation and relaxation (right) is shown. Note that particles identified as being boundary points are not permuted.

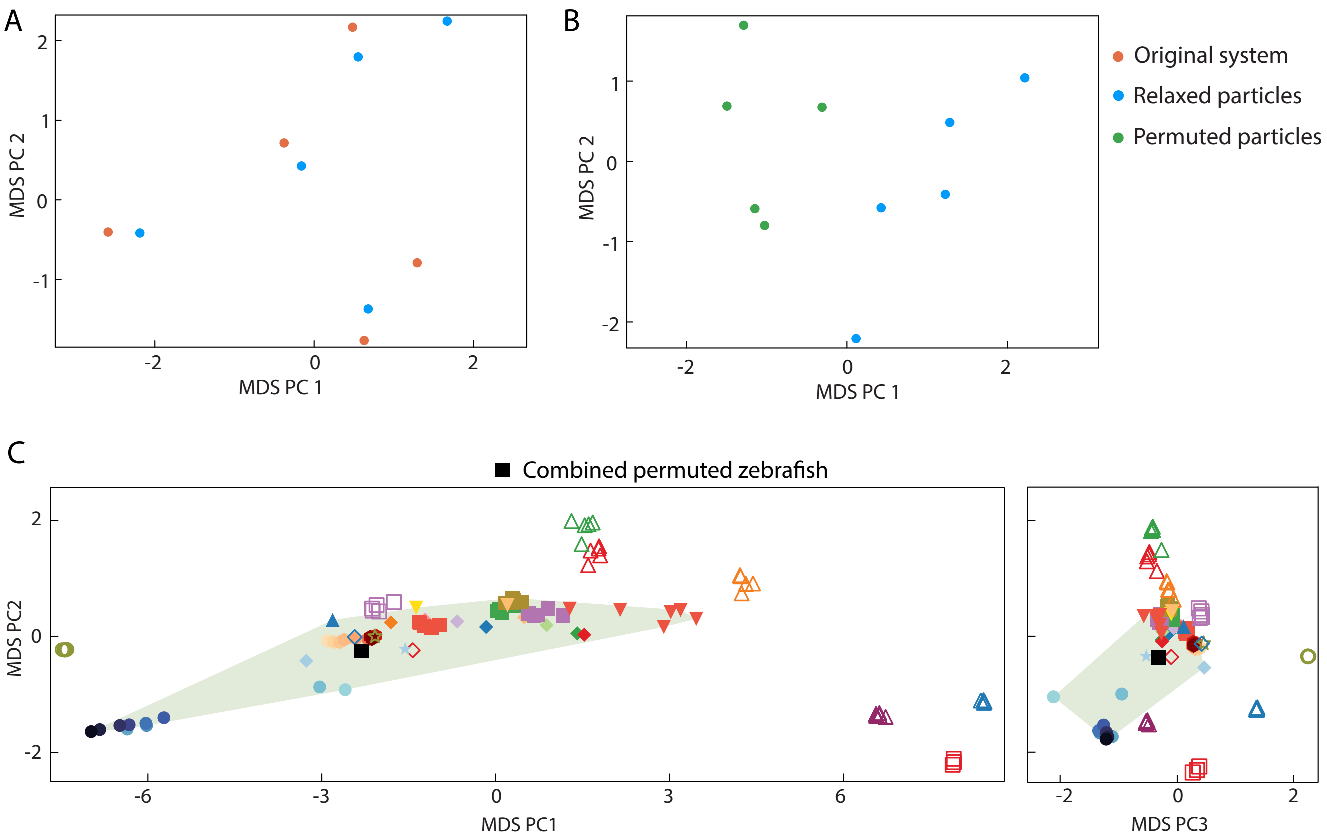

FIG. S21. Permutation of particle positions, and hence removal of growth memory, systematically affects the topological distribution. (A) Relaxing from the initial experimental particle location does not systematically change the topological properties. MDS embedding of the 5 zebrafish experiments before and after relaxation shows the relaxed distributions are close to the topological distribution of the original system before relaxation. (B) Comparing the relaxed points to the permuted points in a 2D MDS embedding finds a systematic difference, with the first principal component distinguishing the two conditions. (C) Combining all experiments for the permuted system and adding to the topological atlas, the permuted system (black square) lies within the living region, but is closest in distance to the industrial foam (for legend see Fig. 3 main text)

VII. TABLES

TABLE S1: Parameter summary for combined topological embedding

Region	Num. samples	Num. points	Boundary protocol	Data source
Bacterial biofilms				
V. cholerae	5	3 combined exp. with $\sim$ 3 time points each with $\sim$ 2000 cells	$\alpha = 4\mu m$	Ref. [32]
E. coli	5	—"—	—"—	—"—
S. enterica	5	—"—	—"—	—"—
P. aeruginosa	5	—"—	—"—	—"—
Zebrafish brain region				
Olfactory Epithelium	1	5 combined exp. each with $\sim$ 1000 cells	$\alpha = 30\mu$m	Ref. [13]
Telencephalon	1	5 combined exp. each with $\sim$ 4000 cells	—"—	—"—
Diencephalon	1	5 combined exp. each with $\sim$ 7000 cells	—"—	—"—
Hypothalamus	1	5 combined exp. each with $\sim$ 2500 cells	—"—	—"—
Mesencephalon	1	5 combined exp. each with $\sim$ 18000 cells	—"—	—"—
Metencephalon	1	5 combined exp. each with $\sim$ 1500 cells	—"—	—"—
Myelencephalon	1	5 combined exp. each with $\sim$ 30000 cells	—"—	—"—
White Matter	1	5 combined exp. each with $\sim$ 5000 cells	—"—	—"—
Spinal Cord	1	5 combined exp. each with $\sim$ 1500 cells	—"—	—"—
Zebrafish embryo				
$t = 625$	1	10 time points from $t = 625$ to $t = 640$, $\sim$ 10,000 cells each	$\alpha = 60\mu m$	Ref. [19]
$t = 775$	1	10 time points from $t = 775$ to $t = 790$, $\sim$ 13,800 cells each	—"—	—"—
$t = 925$	1	10 time points from $t = 925$ to $t = 940$, $\sim$ 15,100 cells each	—"—	—"—
$t = 1075$	1	10 time points from $t = 1075$ to $t = 1090$, $\sim$ 14,800 cells each	—"—	—"—
$t = 1225$	1	10 time points from $t = 1125$ to $t = 1140$, $\sim$ 15,000 cells each	—"—	—"—
$t = 1375$	1	10 time points from $t = 1375$ to $t = 1390$, $\sim$ 15,100 cells each	—"—	—"—
D. melanogaster embryo	14	evenly spaced time points from $t = 270$ to $t = 630$ m.p.f. Contains growing cell numbers from 11,593 to 27,026	$\alpha = 80\mu m$	Ref. [12]
P. mammillata embryo	1	145 frames containing 250-700 cells across 5 experiments	$\alpha = 80\mu$m	Ref. [40]
C. elegans embryo	1	6 experiments at $\sim$350 cell stage	$\alpha = 8\mu$m	Ref. [39]
Human cancer organoid	1	16 experiments at $\sim$200 cell stage	$\alpha = 50\mu$m	Ref. [41]
Glassy dynamics	3	4096	periodic	Ref. [23]
Open-cell polyurethane foam	1	18,000 pores	$\alpha = 300\mu m$	Ref. [43]
Fluid foam simulation	1	21,000 bubbles	$\alpha = 0.06$ a.u.	Ref. [44]
Arabidopsis thaliana apical meristem	6	20 time points each containing $\sim 2,000$ cells,	$\alpha = 20\mu m$	Ref. [42]
Snowflake yeast	1	20 time points each containing $\sim$ 100 cells,	$\alpha = 25\mu m$	Ref. [10]
Random packings				
Sphere packing	5	10,000	periodic	Simulated using Ref. [22]
1:1:4 ellipsoid packing	5	10,000	periodic	—"—
1:4:4 ellipsoid packing	5	10,000	periodic	—"—
1:2:3 ellipsoid packing	5	10,000	periodic	—"—
Polydisperse packing	5	10,000	periodic	—"—
Misc.				
Poisson-Voronoi	5	10,000	periodic	This study

Diffusion limited aggregation	5	10,000	$\alpha = 6.5$ a.u.	Simulated with Ref. [62]
Star survey data	1	$110,000$	$\alpha = 20$ parsecs	Refs [30,63-65]

REFERENCES AND NOTES

1. G. Carlsson, Topology and data. *Bull. Am. Math. Soc.* **46**, 255–308 (2009).

2. W. Bialek, A. Cavagna, I. Giardina, T. Mora, E. Silvestri, M. Viale, A. M. Walczak, Statistical mechanics for natural flocks of birds. *Proc. Natl. Acad. Sci. U.S.A.* **109**, 4786–4791 (2012).

3. N. I. Petridou, B. Corominas-Murtra, C.-P. Heisenberg, E. Hannezo, Rigidity percolation uncovers a structural basis for embryonic tissue phase transitions. *Cell* **184**, 1914–1928.e19 (2021).

4. Y. Katz, K. Tunstrøm, C. C. Ioannou, C. Huepe, I. D. Couzin, Inferring the structure and dynamics of interactions in schooling fish. *Proc. Natl. Acad. Sci. U.S.A.* **108**, 18720–18725 (2011).

5. R. D. MacPherson, D. J. Srolovitz, The von Neumann relation generalized to coarsening of three-dimensional microstructures. *Nature* **446**, 1053–1055 (2007).

6. E. A. Lazar, J. K. Mason, R. D. MacPherson, D. J. Srolovitz, Complete topology of cells, grains, and bubbles in three-dimensional microstructures. *Phys. Rev. Lett.* **109**, 095505 (2012).

7. D.-S. Lee, J. Park, K. A. Kay, N. A. Christakis, Z. N. Oltvai, and A.-L. Barabási, The implications of human metabolic network topology for disease comorbidity. *Proc. Natl. Acad. Sci. U.S.A.* **105**, 9880–9885 (2008).

8. S. H. Strogatz, Exploring complex networks. *Nature* **410**, 268–276 (2001).

9. E. A. Lazar, J. Lu, C. H. Rycroft, Voronoi cell analysis: The shapes of particle systems. *Am. J. Phys.* **90**, 469–480 (2022).

10. T. C. Day, S. S. Höhn, S. A. Zamani-Dahaj, D. Yanni, A. Burnetti, J. Pentz, A. R. Honerkamp-Smith, H. Wioland, H. R. Sleath, W. C. Ratcliff, R. E. Goldstein, P. J. Yunker, Cellular organization in lab-evolved and extant multicellular species obeys a maximum entropy law. *eLife* **11**, e72707 (2022).

11. F. H. Stillinger, T. A. Weber, Packing structures and transitions in liquids and solids. *Science* **225**, 983–989 (1984).

12. P. J. Keller, A. D. Schmidt, A. Santella, K. Khairy, Z. Bao, J. Wittbrodt, E. H. K. Stelzer, Fast, high-contrast imaging of animal development with scanned light sheet–Based structured-illumination microscopy. *Nat. Methods* **7**, 637–642 (2010).

13. Y. Ding, D. J. Vanselow, M. A. Yakovlev, S. R. Katz, A. Y. Lin, D. P. Clark, P. Vargas, X. Xin, J. E. Copper, V. A. Canfield, K. C. Ang, Y. Wang, X. Xiao, F. De Carlo, D. B. van Rossum, P. La Riviere, K. C. Cheng, Computational 3d histological phenotyping of whole zebrafish by x-ray histotomography. *eLife* **8**, e44898 (2019).

14. S. Mu, S.-C. Yu, N. L. Turner, C. E. McKellar, S. Dorkenwald, F. Collman, S. Koolman, M. Moore, S. Morejohn, B. Silverman, K. Willie, R. Willie, D. Bland, A. Burke, Z. Ashwood, K. Luther, M. Castro, O. Ogedengbe, W. Silversmith, J. Wu, A. Halageri, T. Macrina, N. Kemnitz, M. Murthy, H. S. Seung, 3D reconstruction of cell nuclei in a full drosophila brain. bioRxiv 2021.11.04.467197 [**Preprint**]. 4 November 2021. https://doi.org/10.1101/2021.11.04.467197.

15. A. G. Fletcher, M. Osterfield, R. E. Baker, S. Y. Shvartsman, Vertex models of epithelial morphogenesis. *Biophys. J.* **106**, 2291–2304 (2014).

16. L. A. Lardon, B. V. Merkey, S. Martins, A. Dötsch, C. Picioreanu, J.-U. Kreft, B. F. Smets, idynomics: Next-generation individual-based modelling of biofilms. *Environ. Microbiol.* **13**, 2416–2434 (2011).

17. T. Sharp, M. Merkel, M. L. Manning, A. Liu, Inferring statistical properties of 3D cell geometry from 2D slices. *PLOS ONE* **14**, e0209892 (2019).

18. W. Yan, S. Ansari, A. Lamson, M. A. Glaser, R. Blackwell, M. D. Betterton, M. Shelley, Toward the cellular-scale simulation of motor-driven cytoskeletal assemblies. *eLife* **11**, e74160 (2022).

19. P. J. Keller, A. D. Schmidt, J. Wittbrodt, E. H. Stelzer, Reconstruction of zebrafish early embryonic development by scanned light sheet microscopy. *Science* **322**, 1065–1069 (2008).

20. K. McDole, L. Guignard, F. Amat, A. Berger, G. Malandain, L. A. Royer, S. C. Turaga, K. Branson, P. J. Keller, In toto imaging and reconstruction of post-implantation mouse development at the single-cell level. *Cell* **175**, 859–876.e33 (2018).

21. H. Yang, A. F. Pegoraro, Y. Han, W. Tang, R. Abeyaratne, D. Bi, M. Guo, Configurational fingerprints of multicellular living systems. *Proc. Natl. Acad. Sci. U.S.A.* **118**, e2109168118 (2021).

22. A. Donev, I. Cisse, D. Sachs, E. A. Variano, F. H. Stillinger, R. Connelly, S. Torquato, and P. M. Chaikin, Improving the density of jammed disordered packings using ellipsoids. *Science* **303**, 990–993 (2004).

23. V. Bapst, T. Keck, A. Grabska-Barwínska, C. Donner, E. D. Cubuk, S. S. Schoenholz, A. Obika, A. W. R. Nelson, T. Back, D. Hassabis, P. Kohli, Unveiling the predictive power of static structure in glassy systems. *Nat. Phys.* **16**, 448–454 (2020).

24. M. R. Krumholz, C. F. McKee, J. Bland-Hawthorn, Star clusters across cosmic time. *Ann. Rev. Astro. Astro.* **57**, 227–303 (2019).

25. M. Weber, N. Scherf, A. M. Meyer, D. Panáková, P. Kohl, J. Huisken, Cell-accurate optical mapping across the entire developing heart. *eLife* **6**, e28307 (2017).

26. R. van Drongelen, T. Vazquez-Faci, T. A. Huijben, M. van der Zee, T. Idema, Mechanics of epithelial tissue formation. *J. Theor. Biol.* **454**, 182–189 (2018).

27. R. Hartmann, P. K. Singh, P. Pearce, R. Mok, B. Song, F. Díaz-Pascual, J. Dunkel, K. Drescher, Emergence of three-dimensional order and structure in growing biofilms. *Nat. Phys.* **15**, 251–256 (2019).

28. B. Qin, C. Fei, A. A. Bridges, A. A. Mashruwala, H. A. Stone, N. S. Wingreen, B. L. Bassler, Cell position fates and collective fountain flow in bacterial biofilms revealed by light-sheet microscopy. *Science* **369**, 71–77 (2020).

29. E. A. Lazar, J. Han, D. J. Srolovitz, Topological framework for local structure analysis in condensed matter. *Proc. Natl. Acad. Sci. U.S.A.* **112**, E5769–E5776 (2015).

30. W. Gliese, H. Jahreiß, Preliminary version of the third catalogue of nearby stars, in *The Astronomical Data Center CD-ROM: Selected Astronomical Catalogs*, Vol. I; L.E. Brotzmann, S.E. Gesser, Eds. (NASA/Astronomical Data Center, Goddard Space Flight Center, 1991).

31. D. J. Skinner, B. Song, H. Jeckel, E. Jelli, K. Drescher, J. Dunkel, Topological metric detects hidden order in disordered media. *Phys. Rev. Lett.* **126**, 048101 (2021).

32. H. Jeckel, F. Díaz-Pascual, D. J. Skinner, B. Song, E. Jiménez-Siebert, K. Strenger, E. Jelli, S. Vaidya, J. Dunkel, K. Drescher, Shared biophysical mechanisms determine early biofilm architecture development across different bacterial species. *PLoS Biol.* **20**, e3001846 (2022).

33. H. Edelsbrunner, D. Kirkpatrick, R. Seidel, On the shape of a set of points in the plane. *IEEE Trans. Inform. Theory* **29**, 551–559 (1983).

34. I. Borg, P. J. F. Groenen, *Modern Multidimensional Scaling: Theory and Applications* (Springer Series in Statistics, Springer Science & Business Media, ed. 2, 2005), chap. 12, pp. 201–268.

35. P. Pearce, B. Song, D. J. Skinner, R. Mok, R. Hartmann, P. K. Singh, H. Jeckel, J. S. Oishi, K. Drescher, J. Dunkel, Flow-induced symmetry breaking in growing bacterial biofilms. *Phys. Rev. Lett.* **123**, 258101 (2019).

36. V. Alba, J. E. Carthew, R. W. Carthew, M. Mani, Global constraints within the developmental program of the Drosophila wing. *eLife* **10**, e66750 (2021).

37. C. W. Misner, K. S. Thorne, J. A. Wheeler, *Gravitation* (W. H. Freeman and Co., 2000), 23rd printing.

38. L. Liberti, C. Lavor, Six mathematical gems from the history of distance geometry. *Inter. Trans. Operational Res.* **23**, 897–920 (2016).

39. J. Cao, G. Guan, V. W. S. Ho, M.-K. Wong, L.-Y. Chan, C. Tang, Z. Zhao, H. Yan, Establishment of a morphological atlas of the caenorhabditis elegans embryo using deep-learning-based 4d segmentation. *Nat. Commun.* **11**, 6254 (2020).

40. L. Guignard, U.-M. Fíuza, B. Leggio, J. Laussu, E. Faure, G. Michelin, K. Biasuz, L. Hufnagel, G. Malandain, C. Godin, P. Lemaire, Contact area–Dependent cell communication and the morphological invariance of ascidian embryogenesis. *Science* **369**, eaar5663 (2020).

41. Y. L. Han, A. F. Pegoraro, H. Li, K. Li, Y. Yuan, G. Xu, Z. Gu, J. Sun, Y. Hao, S. K. Gupta, Y. Li, W. Tang, H. Kang, L. Teng, J. J. Fredberg, M. Guo, Cell swelling, softening and invasion in a three-dimensional breast cancer model. *Nat. Phys.* **16**, 101–108 (2020).

42. L. Willis, Y. Refahi, R. Wightman, B. Landrein, J. Teles, K. C. Huang, E. M. Meyerowitz, H. Jönssonn, Cell size and growth regulation in the Arabidopsis thaliana apical stem cell niche. *Proc. Natl. Acad. Sci. U.S.A.* **113**, E8238–E8246 (2016).

43. L. Bogunia, S. Buchen, K. Weinberg, Microstructure characterization and stochastic modeling of open-cell foam based on µct-image analysis. *GAMM-Mitteilungen* **45**, e202200018 (2022).

44. P. Karnakov, S. Litvinov, P. Koumoutsakos, Computing foaming flows across scales: From breaking waves to microfluidics. *Sci. Adv.* **8**, eabm0590 (2022).

45. D. Volfson, S. Cookson, J. Hasty, L. S. Tsimring, Biomechanical ordering of dense cell populations. *Proc. Natl. Acad. Sci. U.S.A.* **105**, 15346–15351 (2008).

46. M. A. Heinrich, R. Alert, A. E. Wolf, A. Košmrlj, D. J. Cohen, Self-assembly of tessellated tissue sheets by expansion and collision. *Nat. Commun.* **13**, 4026 (2022).

47. M. J. Crompton, T. M. Dexter, N. A. Wright, C. Hagios, A. Lochter, M. J. Bissell, Tissue architecture: The ultimate regulator of epithelial function? *Phil. Trans. Royal Soc. B* **353**, 857–870 (1998).

48. J. C. Wortman, T.-F. He, S. Solomon, R. Z. Zhang, A. Rosario, R. Wang, T. Y. Tu, D. Schmolze, Y. Yuan, S. E. Yost, X. Li, H. Levine, G. Atwal, P. P. Lee, C. C. Yu, Spatial distribution of b cells and lymphocyte clusters as a predictor of triple-negative breast cancer outcome. *npj Breast Cancer* **7**, 84 (2021).

49. G. Rossi, A. Manfrin, M. P. Lutolf, Progress and potential in organoid research. *Nat. Rev. Genet.* **19**, 671–687 (2018).

50. P. Mehta, M. Bukov, C.-H. Wang, A. G. Day, C. Richardson, C. K. Fisher, D. J. Schwab, A high-bias, low-variance introduction to machine learning for physicists. *Phys. Rep.* **810**, 1–124 (2019).

51. Universal topological atlas, www.topologicalatlas.org.

52. F. Aurenhammer, R. Klein, D.-T. Lee, *Voronoi Diagrams and Delaunay Triangulations* (World Scientific Publishing Co. Inc., ed. 1, 2013).

53. J. Solomon, R. Rustamov, L. Guibas, A. Butscher, Earth mover's distances on discrete surfaces. *ACM Trans. Graph.* **33**, 1–12 (2014).

54. H. Edelsbrunner, J. Harer, Persistent homology-a survey. *Cont. Math.* **453**, 257 (2008).

55. M. Čufar, Ripserer.jl: Flexible and efficient persistent homology computation in julia. *J. Open Source Soft.* **5**, 2614 (2020).

56. J. Solomon, R. Rustamov, L. Guibas, A. Butscher, Continuous-flow graph transportation distances. arXiv:1603.06927 [cs.OH] (22 March 2016).

57. E. Saucan, A. Samal, J. Jost, A simple differential geometry for complex networks. *Network Sci.* **9**, S106–S133 (2021).

58. S. Chewi, J. Clancy, T. Le Gouic, P. Rigollet, G. Stepaniants, A. Stromme, Fast and smooth interpolation on wasserstein space. *Proc. Mach. Learn. Res.* **130**, 3061–6069 (2021).

59. L. Hall-Stoodley, J. W. Costerton, and P. Stoodley, Bacterial biofilms: From the natural environment to infectious diseases. *Nat. Rev. Microbiol.* **2**, 95–108 (2004).

60. H. C. Flemming, S. Wuertz, Bacteria and archaea on Earth and their abundance in biofilms. *Nat. Rev. Microbiol.* **17**, 247–260 (2019).

61. T. A. Witten, L. M. Sander, Diffusion-limited aggregation. *Phys. Rev. B* **27**, 5686–5697 (1983).

62. M. Fogleman, Diffusion limited aggregation (2019); https://github.com/fogleman/dlaf.

63. D. Nash, The hyg database (2014).

64. D. Hoffleit, J. Warren, W. H. VizieR Online Data Catalog: Bright Star Catalogue, 5th Revised Ed. (Hoffleit+, 1991), *VizieR Online Data Catalog* (1995).

65. European Space Agency, Vol. 1200 (ESA Publications Division, 1997).

66. F. Beroz, J. Yan, Y. Meir, B. Sabass, H. A. Stone, B. L. Bassler, N. S. Wingreen, Verticalization of bacterial biofilms. *Nat. Phys.* **14**, 954–960 (2018).

67. J.-F. Ferellec, G. R. McDowell, A method to model realistic particle shape and inertia in dem. *Granular Matt.* **12**, 459–467 (2010).
